# Supplementary material for: Built Environments And Child Health in WalEs and AuStralia (BEACHES): a study protocol
Source: BMJ Open. 2022 Oct 25;12(10):e061978. doi: 10.1136/bmjopen-2022-061978 (PMC9608521; doi:10.1136/bmjopen-2022-061978)
Supplement: Author's manuscript [file bmjopen-2022-061978.draft_revisions.pdf]

BMJ Open

BMJ Open is committed to open peer review. As part of this commitment we make the peer review history of every article we publish publicly available.

When an article is published we post the peer reviewers' comments and the authors' responses online. We also post the versions of the paper that were used during peer review. These are the versions that the peer review comments apply to.

The versions of the paper that follow are the versions that were submitted during the peer review process. They are not the versions of record or the final published versions. They should not be cited or distributed as the published version of this manuscript.

BMJ Open is an open access journal and the full, final, typeset and author-corrected version of record of the manuscript is available on our site with no access controls, subscription charges or pay-per-view fees (http://bmjopen.bmj.com).

If you have any questions on BMJ Open's open peer review process please email info.bmjopen@bmj.com

BMJ Open

Built Environment and Child Health in Wales and Australia (BEACHES): a study protocol

Journal:	*BMJ Open*
Manuscript ID	bmjopen-2022-061978
Article Type:	Protocol
Date Submitted by the Author:	14-Feb-2022
Complete List of Authors:	Pedrick-Case, Rebecca; Swansea University Medical School, Population Data Science<br>Bailey, Rowena; Swansea University Medical School, Population Data Science<br>Beck, Ben; Monash University, School of Public Health and Preventive Medicine<br>Beesley, Bridget; The University of Western Australia, UWA School of Agriculture and Environment<br>Boruff, Bryan; The University of Western Australia, UWA School of Agriculture and Environment<br>Brophy, Sinead; Swansea University Medical School, Population Data Science<br>Cross, Donna; The University of Western Australia, Telethon Kids Institute; The University of Western Australia, School of Population and Global Health<br>Dhamrait, Gursimran; The University of Western Australia, Telethon Kids Institute; The University of Western Australia, School of Population and Global Health<br>Duncan, John; University of Western Australia, UWA School of Agriculture and Environment<br>Gething, Peter; The University of Western Australia, Telethon Kids Institute; Curtin University, Faculty of Health Sciences<br>Johnson, Rhodri; Swansea University Medical School, Population Data Science<br>Lyons, Ronan; Swansea University Medical School, Population Data Science<br>Mizen, Amy; Swansea University Medical School, Population Data Science<br>Murray, Kevin; University of Western Australia, School of Population and Global Health<br>Pouliou, Theodora; Swansea University Medical School, Population Data Science<br>Rafferty, James; Swansea University Medical School, Population Data Science<br>Rosenberg, Michael; University of Western Australia, School of Human Sciences<br>Schipperijn, Jasper; University of Souther Denmark, Department of Sports Science and Clinical Biomechanics<br>Thompson, Daniel; Swansea University Medical School, Population Data Science<br>Trost, Stewart; The University of Queensland, School of Human

	Movement and Nutrition Sciences<br>Watkins, Alan; Swansea University Medical School<br>Stratton, Gareth; Swansea University, Research Centre in Applied Sports, Technology, Exercise and Medicine<br>Fry, Richard; Swansea University Medical School, Population Data Science<br>Christian, Hayley; The University of Western Australia, Telethon Kids Institute; The University of Western Australia, School of Population and Global Health<br>Griffiths, Lucy ; Swansea University, Swansea University Medical School
Keywords:	EPIDEMIOLOGY, PAEDIATRICS, PREVENTIVE MEDICINE, PUBLIC HEALTH

SCHOLARONE™
Manuscripts

Built Environment and Child Health in Wales and Australia (BEACHES): a study protocol

Rebecca Pedrick-Case[1], Rowena Bailey[1], Ben Beck[2], Bridget Beesley[3], Bryan Boruff[3], Sinead Brophy[1], Donna Cross [4,5], Gursimran Dhamrait [4,5], John Duncan[3], Peter Gething[4,6], Rhodri D Johnson[1], Ronan A Lyons[1], Amy Mizen[1], Kevin Murray[5], Theodora Pouliou[1], James Rafferty[1] Michael Rosenberg[7], Jasper Schipperijn[8], Daniel A Thompson[1], Stewart Trost[9], Alan Watkins[1], Gareth Stratton[10, #], Richard Fry[1,#], Hayley Christian [4,5,#], Lucy J Griffiths[1,#,*]

[1] Population Data Science, Swansea University Medical School, Swansea, UK.

[2] School of Public Health and Preventive Medicine, Monash University, Melbourne, Australia.

[3] UWA School of Agriculture and Environment, University of Western Australia, Perth, Australia.

[4] Telethon Kids Institute, University of Western Australia, Perth, Australia.

[5] School of Population and Global Health, University of Western Australia, Perth, Australia.

[6] Faculty of Health Sciences, Curtin University, Perth, Australia.

[7] School of Human Sciences, University of Western Australia, Perth, Australia.

[8] Department of Sports Science and Clinical Biomechanics, University of Southern Denmark, Odense, Denmark.

[9] School of Human Movement and Nutrition Sciences, University of Queensland, Brisbane, Australia.

[10] Research Centre in Applied Sports, Technology, Exercise and Medicine (A-STEM), Swansea University, Swansea, UK.

* Corresponding author: Dr Lucy Griffiths, Swansea University Medical School, Swansea, University, Swansea, UK; lucy.griffiths@swansea.ac.uk

# Joint senior authors

ORCID IDs

For peer review only

Rebecca Pedrick-Case: 0000-0003-2307-1808

Rowena Bailey: 0000-0003-2409-2045

Ben Beck: 0000-0003-3262-5956

Bridget Beesley: 0000-0003-0432-3766

Bryan Boruff: 0000-0001-6693-0671

Sinead Brophy: 0000-0001-7417-2858

Donna Cross: 0000-0001-6217-5058

Gursimran K Dhamrait: 0000-0002-5191-211X

John Duncan: 0000-0001-9752-1002

Peter Gething: 0000-0001-6759-5449

Rhodri D Johnson: 0000-0001-9636-0753

Ronan A Lyons: 0000-0001-5225-000X

Amy Mizen: 0000-0001-7516-6767

Kevin Murray: 0000-0002-8856-6046

Theodora Pouliou: 0000-0002-1162-1174

James Rafferty: 0000-0002-1667-7265

Michael Rosenberg: 0000-0002-9950-5775

Jasper Schipperijn: 0000-0002-6558-7610

Daniel A Thompson: 0000-0002-1769-2870

Stewart Trost: 0000-0001-9587-3944

Alan Watkins: 0000-0003-3804-1943

Rich Fry: 0000-0002-7968-6679

Gareth Stratton: 0000-0001-5618-0803

Hayley Christian: 0000-0001-8486-5746

Lucy J Griffiths: 0000-0001-9230-624X

Word Count: 3568/4,000 words

Keywords: Non-communicable disease, childhood obesity, built environment, linked data, cohort studies, ORIGINS Project, PLAYCE Cohort Study, Longitudinal Study of Australian Children, Millennium Cohort Study, SAIL Databank.

For peer review only

Abstract

Introduction: Childhood obesity and physical inactivity are two of the most significant modifiable risk factors for the prevention of non-communicable diseases (NCDs). Yet, a third of children in Wales and Australia are overweight or obese, and only 20% of UK and Australian children are sufficiently active. The purpose of this study is to identify and understand how complex and interacting factors in the built environment influence modifiable risk factors for NCDs across childhood.

Methods & Analysis: This is an observational study using data from five established cohorts from Wales and Australia: 1) Wales Electronic Cohort for Children (WECC); 2) Millennium Cohort Study (MCS); 3) PLAY Spaces & Environments for Children's Physical Activity (PLAYCE) study; 4) the ORIGINS Project; and 5) The Growing Up in Australia: the Longitudinal Study of Australian Children (LSAC). The study will incorporate a comprehensive suite of longitudinal quantitative data (surveys, anthropometry, accelerometery, and Geographic Information Systems (GIS) data) to understand how the built environment influences children's modifiable risk factors for NCDs (Body Mass Index (BMI), physical activity, sedentary behaviour, and diet).

Ethics & Dissemination: This study has received the following approvals: The University of Western Australia Human Research Ethics Committee (2020/ET000353), Ramsay Human Research Ethics Committee (under review), and Swansea University Information Governance Review Panel approval (Project ID: 1001). Findings will be reported to the following: 1) funding bodies, research institutes and hospitals supporting the BEACHES project; 2) parents and children; 3) existing and new industry partner networks; 4) federal, state, and local governments to inform policy; as well as 5) presented at local, national, and international conferences; and 6) disseminated by peer-review publications.

For peer review only

For peer review only - http://bmjopen.bmj.com/site/about/guidelines.xhtml

Article Summary

Strengths and limitations of this study

- The BEACHES project uses large representative samples of children from five Wales and Australian cohort studies to identify and understand how complex and interacting factors in the built environment influence modifiable risk factors for NCDs across childhood.
- Standardised built environment measures will be applied to five Welsh and Australian cohort datasets, representing more than 1 million children.
- The use of existing cohort datasets can limit the use of consistent health outcomes across studies.
- The contrasting timepoints, climates, geographies, and policy approaches in Wales and Australia will provide stronger evidence of the causal pathways between the built environment and child health ultimately leading to scientifically robust policy recommendations.

Introduction

Childhood obesity and physical inactivity are two of the most significant modifiable risk factors for non-communicable disease (NCD) prevention in children.[1] The 2017 Commission on Ending Childhood Obesity emphasised that the prevention of modifiable risk factors for NCDs should start as early as possible.[2] Yet only 20% of UK and Australian children are sufficiently active, and over 60% engage in excessive sedentary time, with a third overweight or obese.[3]

The built environment in which we live is integral to human health. Research has shown that residing in 'liveable' neighbourhoods characterised by: good access to shops, services, quality parks, connected streets to facilitate walking, sufficient residential densities to support public transport services and local businesses, minimal crime and traffic, and social connectedness opportunities are associated with improved health outcomes in adults.[4–7] Despite the increasing evidence of the association between the built environment and physical activity in adults, there is a paucity of longitudinal research examining the role of the built environment in promoting child health.[8,9] Critical evidence gaps include: 1) causal evidence of the overall impact the built environment has on children's modifiable risk factors for NCDs (i.e., physical inactivity, sedentariness, and poor diet);[9,10] 2) the specific effect of individual, familial, and combinations of built environment attributes; 3) how these

For peer review only - http://bmjopen.bmj.com/site/about/guidelines.xhtml

effects vary across different ages of children;[11] 4) how these effects are moderated by socioeconomic status;[12] 5) the mediating role of physical activity and healthy eating on the relationship between the built environment and child obesity;[13] and 6) how the influence of the built environment on children's modifiable risk factors for NCDs varies by different geographical locations.[14] These evidence gaps hinder the formulation of specific, actionable policies to improve the built environment for child health.

The Built Environment and Child Health in Wales and Australia (BEACHES) project is a collaboration between academic institutions in Wales and Australia to identify and understand how complex and interacting factors in the built environment influence modifiable risk factors for NCDs across childhood. This project aims to address identified evidence gaps by using Wales and Australian longitudinal population linked and cohort data to identify and understand how complex and interacting factors in the built environment influence modifiable risk factors for NCDs (physical inactivity, sedentariness, poor diet) across childhood. Five large Welsh and Australian cohort studies, with detailed anthropometric, physical (in)-activity, diet, and contextual data for more than one million children will be used. We will use the highest quality available spatial data and geospatial techniques to construct an internationally standardised set of metrics, such as walkability, that characterise the built environments each child has inhabited during childhood. A statistical modelling framework will be used to quantify the influence that different built environment characteristics have on children’s BMI, and the respective roles of physical activity, sedentary behaviour, and diet in this relationship.

Objectives

The purpose of this study is to identify and understand how complex and interacting factors in the built environment influence modifiable risk factors for NCDs across childhood. The objectives are to:

1. Develop a comprehensive Geographic Information Systems (GIS) model of child-specific built environment characteristics using standardised methods for Wales and Australia,
2. Link standardised GIS models of the built environment to e-cohort and standard cohort data for children in Wales and Australia,
3. Determine the direct and indirect (and mediating) relationships between the built environment and: a) children’s modifiable risk factors for NCDs (physical activity, sedentary time, and diet), and b) obesity,
4. Identify how relationships between the built environment and these NCD risk factors vary by

For peer review only - http://bmjopen.bmj.com/site/about/guidelines.xhtml

children's socio-economic position and geographic location (across and within Wales and Australia), and

5. Produce evidence which policymakers and other stakeholders can use to modify the built environment to reduce childhood obesity and NCDs.

Methods and analysis

This multi-site study will use population-level cohort data (including but not limited to surveys, anthropometry, accelerometry, GIS data, and biological samples) to address these five objectives (Figure 1). We will review the policy landscape and literature on the key built environment and child health-related policies at national and local levels in Wales and Australia. Based on the findings, we will curate and harmonise environmental data that will be used to develop high resolution spatial models of the built environment. These built environment measures will be linked with cohort and population-level health data (Wales) to identify and understand how complex and interacting factors in the built environment influence modifiable risk factors for NCDs and BMI across childhood using a series of covariate-adjusted, multi-level regression models. Finally, we will communicate to policymakers and planners which modifiable aspects of the built environment may contribute to a reduction in risk factors for NCDs and BMI across childhood.

INSERT Figure 1

Policy & Literature Review

We will discuss our project with stakeholders and carry out a policy landscape review (for the duration of the cohort studies) to examine key built environment and child health-related policies at national and local levels in Wales and Australia. Findings will provide the overall context for policy gaps and landscapes, and the co-design of research to address policy gaps, develop best practice guidelines and overall, reduce NCDs in children.

Our stakeholders include health service commissioners, government departments of planning, transport and health, planning officials, urban planners, building industry, developers and key advocacy NGOs (non-governmental organisations), such as the British Heart Foundations and Active Healthy Kids Wales as well as third sector and voluntary agencies.

For peer review only

In Wales, we will engage with our stakeholders at three time points throughout the project using appropriate frameworks (e.g. 'appreciative inquiry') [15,16] to inform and interpret our quantitative research findings into stakeholder engagement activities. We will map out policies and key legislation across Wales using the six priority areas of the built and natural environment that Public Health Wales has published in the 'Creating healthier places and spaces for our present and future generations 'report.[17]

In Australia, our government and non-government partners include the Western Australian (WA) Department of Local Government, Sport and Cultural Industries, WA Department of Health, WA Department of Transport, WA Local Government Association, Australian Childcare Alliance, Nature Play Australia, Heart Foundation, The PLAY Spaces and Environments for Children's Physical Activity (PLAYCE) partners, Cancer Council WA, Goodstart Early Learning, and Hames Sharley. With our parterns, we will conduct a policy analysis to investigate how Western Australian and national policies address the health of children through the built environment's influence on obesity and the modifiable risk factors for obesity, physical activity, sedentary behaviour, and diet. Policy analysis is crucial to achieving reforms in health promotion by raising awareness of current policy gaps and opportunities and demonstrating successful policy related actions being taken across the system. The Comprehensive Analysis of Policy on Physical Activity (CAPPA) framework will be used to guide the analysis.[18]

Built Environment Measures

GIS-derived built environment measures will be calculated at the residential address level for Wales and Australia. Table 1 outlines the standardised built environment measures that will be created for all Wales and Australian datasets. We will use rich vector spatial datasets which define land utilisation (e.g. building footprint and height, cadastre, road centreline and reserve, and points of interest), planning data (e.g. active travel routes, open space assessments, fast food outlets) and Earth Observation data (e.g. Landsat, Sentinel, Aerial Photography). We have developed a broad range of longitudinal data and methods which characterise the built environment of the two study areas (Table 2). These include neighbourhood walkability, garden size/home outdoor area, blue-green space availability,[19] access to services and facilities, and fast-food outlets around the home and school.

In previous studies of built-environment measures for physical activity, how the neighbourhood is defined has greatly influenced the results.[20] For example, access to green and blue spaces or destinations (retail and food outlets) can be based on distance from a child's residence measured as a straight-line (Euclidean distance) from a household to a destination or based on movement along a

For peer review only - http://bmjopen.bmj.com/site/about/guidelines.xhtml

211 road network, with often very different results. Therefore, the GIS and epidemiological teams will work together closely with a ‘behaviour-led approach’ to define the neighbourhood sizes most likely to correlate with the behaviour of interest by age group. For example, active travel from home to school.

Harmonisation of Built Environment Measures

As far as reasonably possible, the data, spatial analytic approaches, and built environment measures will be standardised and harmonised across the Welsh and Australian study cohorts to enable the calculation of comparable measures. Standardisation and harmonisation will increase confidence in the comparability of measures by minimising potential errors from heterogeneity in data collection procedures, software or methodological approaches; and is particularly important in projects between two countries where definitions, symbology, and projection in source cartographic data will vary.[21] Where feasible, we will use open data (e.g. OSM) and open-source software, such as QGIS, SQL, Python or R, to reduce barriers to reproducing this research in other contexts, thus increasing the potential impact. Methods and code will be made available in future publications, and variations in terminology will be mapped out in a typology with definitions. This will help us to identify where broader policy approaches can be taken and where more contextually specific policies will need to be implemented.[22]

For peer review only

For peer review only - http://bmjopen.bmj.com/site/about/guidelines.xhtml

Table 1. *Wales and Australian Standardised Measures of the Built Environment.*

Built Environment Feature	Indicative BE Variables	Data Source	
		Wales	Australia
**Outdoor home environment**	Home garden/yard space & vegetation	Aerial imagery, NDVI/EVI	Aerial imagery, NDVI/EVI, Urban Monitor, Landgate (Cadastre)
**Child-relevant walkability**	Road traffic exposure, dwelling density, residence type, street connectivity, one-way node count, land use mix, 'local living score', public transport stops	OS Master Map, OSM	Landgate, Main Roads Western Australia, Western Australian Public Transport Authority, Valuer Generals Office, OSM
**Child-relevant green/blue space**	Public open space type (park, school grounds, natural, club/playgrounds, residual greenspace), park size category (Pocket/small, medium/large, district/regional), park features and attractiveness, vegetation, blue-space (distance to coast)	OS Master Map & Greenspace, OSM	University of Western Australia – Centre for the Built Environment and Health, Landgate
**Child-relevant destinations and features**	Playgroups, childcare centres, before and after school care services, kindergarten/nurseries, primary school, secondary school, other	Local authority data, OSM	Playgroup WA Inc, Western Australian Government Department of Local Government, Sport and Cultural Industries' Department of Education, Department of Transport, Department of Planning, Lands and Heritage, Department of Health
**Socioeconomic status**	Such as household level of income.	WIMD, individual-level SES data in SAIL databank	Socio-Economic Indexes for Areas (SEIFA)
**Household location**	Required to derive other measures	UPRN (OS), linked to RALF within SAIL database.	Property Street Address (PSA), GNAF

NDVI: Normalised Difference Vegetation Index; EVI: Enhanced Vegetation Index, OSM: Open Street Map, OS: Ordnance Survey, ONS: Office for National Statistics, SAIL: Secure Anonymised Information Linkage; UPRN: Unique Property Reference Number, RALF: Residential Anonymised Linking Field, GNAF: Geoscape Geocoded National Address File.

For peer review only - http://bmjopen.bmj.com/site/about/guidelines.xhtml

Data Sources, Linkage and Statistical Analysis (Epidemiology)

Built environment models (see above) will be linked with child cohort data to investigate how complex and interacting factors in the built environments may influence modifiable risk factors for NCD's and BMI across childhood.

Data Sources

Five large Welsh and Australian cohort studies will be used. They include: 1) the Wales Electronic Cohort for Children (WECC); 2) the Millennium Cohort Study (MCS Wales); 3) the PLAY Spaces & Environments for Children's Physical Activity (PLAYCE) study (Australia); 4) the ORIGINS Project (Australia); and 5) The Growing Up in Australia: The Longitudinal Study of Australian Children (LSAC; Table 2). Further details about each of the datasets are provided below. The age range covers both childhood and adolescence, with multiple data collection dates for each cohort study.

Table 2. Overview of Wales and Australian health datasets.

**Characteristics**	**Datasets & Location**				
	**WECC (Wales)**	**MCS (Wales)**	**ORIGINS (AU)**	**PLAYCE (AU)**	**LSAC (AU)**
**Age**	0-17	5-14	1-3	2-9	9-17
**Sample size**	1.3 million	1,699-2,181	2,036	2,028	8,416
**No. time points**	Dynamic	4	2	3	8
**Collection years**	1990-ongoing	2006-2015	2018-2022	2015-2022	2008-2018
**BMI**	⍰*	⍰	⍰	⍰	⍰
**Physical Activity**	X	⍰	⍰	⍰	⍰
**Sedentary time**	X	⍰	⍰	⍰	⍰
**Diet**	X	⍰	⍰	⍰	⍰
**Confounders**	⍰	⍰	⍰	⍰	⍰
**Geolocation**	Home, school	Home, School	Home	Home, childcare, school	Home

UK: United Kingdom, AU: Australia, WECC: Wales Electronic Cohort for Children, MSC: Millennium Cohort Study, PLAYCE: PLAY Spaces and Environments for Children's Physical Activity, LSAC: The Longitudinal Study of Australian Children. *BMI data is available for *n*=250,000 records.

Wales

<u>The Wales Electronic Cohort for Children (WECC)</u>

WECC is a total population anonymised electronic cohort study of all children born and living in Wales since 1990 (Table 2) (n=1.3 million) which is updated every three months. The WECC includes information for all live births (approximately 35,000 births per year), Welsh resident mother at birth, or a child who becomes a resident in Wales as identified by registering with a GP practice in Wales. This will be supplemented with population-level health data (general practitioner records, hospital

For peer review only - http://bmjopen.bmj.com/site/about/guidelines.xhtml

admissions) and BMI data from the Child Measurement Programme in Wales (measured height and weights at age 5 years). This dataset also includes linkage to total population education data and a subset of child reported measures (20,000+) including diet, physical activity/physical literacy, wellbeing, child-assessed rating of local area for safety/play. A fuller description of the WECC and its contents can be found in.[23]

The Millennium Cohort Study (MCS)

The MCS is a multi-disciplinary research project following the lives of children born in the UK between September 2000 and January 2002.[24] It provides information relating to approximately 2,000 children and young people resident in Wales at ages 5, 7, 11 and 14 years. Information includes measured heights and weights using standardised protocols,[25] accelerometery data,[26] and parental-reported measures of health, diet, sedentary behaviour (e.g. parent-report screen time) and physical activity behaviours (e.g. sports club and playing outside),[27,28] as well as a range of covariate information relating to social and demographic factors of the children and their families.

Australia

PLAY Spaces & Environments for Children's Physical Activity (PLAYCE – Western Australia)

The PLAY Spaces & Environments for Children's Physical Activity (PLAYCE) is an ongoing cohort study (2015-current), investigating the influence of the childcare-, home-, and neighbourhood- built environments (parent perceptions and objectively measured) on children's physical activity, sedentary behaviour, diet and weight status.[29] Baseline (Timepoint 1) data were collected for 2,028 children, aged 2-5 years, recruited from 118 childcare centres (response rate 46%) in metropolitan Perth, Western Australia. Childcare centres were recruited evenly across socio-economic statuses and size (small and large). Two additional time points of data have been collected once children commenced formal schooling: 1) Timepoint 2: 5-7 years (2019-20); and 2) Timepoint 3: 7-9 years (2020-21). The study design enables modifiable risk factors to be examined as children transition between different built environment settings: home, childcare; and full-time school. Full details of the PLAYCE study protocol have been published.[30]

Physical activity was measured using accelerometery, and parent-report structured and unstructured physical activity including outdoor play.[30–33] Sedentary behaviour was measured using accelerometery, (time spent sedentary) and parent-report screen time.[30–32] Diet was assessed using a parent-report short food frequency questionnaire.[34] Children's height, weight and waist circumference were measured by the research team using standard protocols.

For peer review only - http://bmjopen.bmj.com/site/about/guidelines.xhtml

The ORIGINS Project (Western Australia)

ORIGINS is a collaborative initiative between the Telethon Kids Institute and Joondalup Health Campus. It is a birth cohort of 10,000 families enrolled during pregnancy and followed children from birth to age five.[35] ORIGINS is fully integrated into clinical and diagnostic services. It aims to improve understanding of the early antecedent pathways that mediate NCDs development through the study of early environments, maternal and paternal physical health, and genetics. ORIGINS overarching goal is to reduce the rising epidemic of NCDs by providing children with 'a healthy start to life.' In 2021, the cohort will include data on 2,036 children collected at 12 and 36 months.

Physical activity was measured using parent-report structured and unstructured physical activity as well as outdoor play.[33] Sedentary behaviour was measured via parent-report screen time. Parents reported their child's diet using the Australian Eating Survey; a validated semi-quantitative food frequency questionnaire.[36] Children's height, weight and waist circumference were measured by trained staff.

Longitudinal Study of Australian Children (LSAC - Australia)

Growing Up in Australia: The Longitudinal Study of Australian Children (LSAC) is an ongoing prospective study that used a two cohort cross-sequential design and in 2004 recruited 5,107 children to the baby cohort (median age = 9 months) and 4,983 children to the kindergarten cohort (median age = 4.8 years).[37] The sampling method provided a representative sample of Australian children. Data were collected every two years for both cohorts. Data from children aged 9-17 years will be included. Full details of the LSAC study are published elsewhere.[37]

Children's physical activity was measured using parent-report time-use diaries which included structured and unstructured physical activity. Sedentary behaviour was gathered through parent reports and the children's time use diary for three types of screen-based activities (television viewing, computer use and electronic games).[38] Each parents reported their child's diet via food diaries. In the Child Health CheckPoint, children completed a questionnaire regarding their usual intake of various foods including fruit and vegetable consumption, water intake, and breakfast consumption. Children's height, weight, body composition, and waist circumference were measured by parents.

Data Linkage

Wales

Routinely collected health data and cohort study data from studies such as the Millennium Cohort Study, are available via the Secure Anonymised Information Linkage (SAIL) databank.[39,40] This is a

For peer review only - http://bmjopen.bmj.com/site/about/guidelines.xhtml

unique resource of linked longitudinal health, socio-economic and environmental data relating to the people of Wales. One of the core linkage mechanisms is the Residential Anonymised Linking Field (RALF).[41] The benefits of the RALF system are two-fold. First, it allows the GIS modelling of population-level socio-environmental measures for approximately 1.4 million households in Wales at ±1m spatial resolution. Second, it allows the grouping of individuals into household units in SAIL; thus, reducing ecological fallacies and the impacts of the modifiable areal unit problem.

Australia

Home addresses will be geocoded for all three Australian cohorts (ORIGINS, PLAYCE and LSAC) to enable the creation of GIS measures of the natural and built environment by the Australian team.[30,42] PLAYCE and ORIGINS cohort data will be linked to built environment measures using a unique participant identification number. LSAC data linkage will involve the Australian Bureau of Statistics, the Australian Institute of Family Studies' Data Linkage and Integrating Authority, the Australian Institute of Family Studies' Data Management team, National Centre for Longitudinal Data.

Statistical analysis

A series of covariate-adjusted, multi-level regression and machine learning models will be fitted to explore the impact of different aspects of the built environment on BMI/NCD risk factors, to quantify the effect of each associative relationship using a common outcome variable. A multi-level model structure will be used to adjust for inherent autocorrelation that exists within groups of individuals. Covariates will include, for example, child age, sex, SES (e.g., parent education, neighbourhood level disadvantage), and family composition. We will also examine how these relationships vary by different population groups (e.g., young children, school age children and adolescents). This series of analyses will enable a comparison and prioritisation of the built environment factors that have the strongest associations with BMI and quantify the magnitude of variation in effect size across various built environment features. We will then standardise these results to rank characteristics of the built environment which may contribute to better health, to assist prioritisation of public services and resources.

The direct, indirect, and total effects of the built environment on children's BMI and other NCD risk factors will be estimated using well established multi-level mediation modelling. We will also determine if differences in these factors between advantaged and disadvantaged neighbourhoods can be explained in part by differences in built environment attributes (e.g., better or poorer access to green space). The mediation effect of physical activity and the moderation effect of neighbourhood socio-economic disadvantage on the relationship between the built environment and BMI will initially

For peer review only - http://bmjopen.bmj.com/site/about/guidelines.xhtml

be tested as separate models. A moderated path analysis using all longitudinal data will combine moderation and mediation, and identify direct, indirect, and total mediation effects, and show how these effects vary across the levels of the moderator. All analyses will adjust for baseline and, where appropriate, temporal confounding. Special attention will also be paid to potential confounders of the outcome (e.g. BMI) and the mediating variable (e.g. physical activity) being examined. The role of age, sex, socio-economic status and urban/rural locality will be examined.

Statistical analyses will be conducted separately for Welsh and Australian datasets. A set of built environment measures will be standardised and linked to the five cohort datasets, however there are small differences in the methodology used in each cohort that makes pooling datasets unfeasible. Findings from both countries' analysis of their respective linked health cohort and built environment datasets will be compared to determine differences between different age groups of children in Wales and Australia.

Patient and Public Involvement

Development of the research questions and outcome measures were informed by ongoing engagement with stakeholders who have an interest in child health, such as Public Health Wales, and the PLAYCE Community Reference Group (Western Australia). This study uses anonymised routinely collected and cohort data, so it was not possible to directly consult the individuals included in the study.

Results will be disseminated by engaging children directly such as via a short video that can be shown in schools and online, science outreach workshops, and existing child-health networks to translate the findings into useful advice for children, their families, and policy makers. Please refer to 'Ethics & Dissemination' below further information.

Ethics & Dissemination

All data will be anonymised and, in Wales, linked within the privacy protecting SAIL Databank. We will be using anonymised data and therefore we are exempt from National Research Ethics Committee (NREC). An Information Governance Review Panel (IGRP) application [Project ID: 1001] to link these data has been approved. The PLAYCE study has existing Human Research Ethics Approval from The

For peer review only

University of Western Australia (UWA: #RA/4/1/7417). An application will be submitted to the Ramsay Human Research Ethics Committee Western Australia for the ORIGINS and built environment analyses and to the UWA Human Research Ethics Committee for the LSAC and built environment secondary analyses. All Australian research will abide by the NHMRC's National Statement on Ethical Conduct in Human Research (2008) and is considered negligible risk.

Our dissemination and impact generation strategies will be informed by research on knowledge mobilization models; [43] to impact at a variety of scales (including national and local government and across multiple sectors) and will not be confined to a Welsh-Australian context. Throughout, we will involve stakeholders and policy makers in the research process from inception to translation, seeking their feedback on our process, progress, and findings. We will disseminate findings to the public at events of interest to parents and children and through existing and new industry partner networks. An information sheet will be prepared in plain English with infographics, and a video explaining the project and its findings will be made available online and advertised through social media. Findings from the study will be published in a comprehensive report and an infographic summary. We will notify all stakeholders and promote publication through international networks established during this research. We will hold seminars and workshops to report our findings to stakeholders and the public.

Discussion and Conclusion

The purpose of this study is to identify and understand how complex and interacting factors in the built environment influence modifiable risk factors for NCDs across childhood. Harmonising both child and built environment indicators across five large-scale studies enables analysis of an effectively larger sample size, spans a broader set of age-ranges, and leverages greater heterogeneity in built environments. By harmonising analyses from Welsh and Australian settings, the BEACHES project provides a unique opportunity to identify impacts of the built environment that are common across settings and where place-specific, physical, cultural, and policy environments mediate these effects.

We aim to communicate to policy and planners which modifiable aspects of the built environment result in the most significant reductions in the risk factors for NCDs across childhood. This will enable action at both a national and international scale and, in turn, will contribute to population-level reductions in NCDs. This is an essential component of our work to able to bring about large-scale

For peer review only - http://bmjopen.bmj.com/site/about/guidelines.xhtml

improvement in the built environment to reduce NCDs beginning in early childhood. Furthermore, the outputs of this research could be used to inform future research by aggregating evidence of individual associations in a single dynamic model.

Contributorship statement

HC, GS, LG, and RF are the Principal Investigators who conceptualised the overall study, established research collaborations and led the project implementation. Co-Is who contributed to obtaining the research funding include SB, AW, RAL, JR, AM, BB, BBK, ST, DC, MR, KM, PG, JS. RF, BB, RP-C, AM, are responsible for the development of Geographic Information Systems measures and associated data analysis. RP-C, GKD, RF, HC, and LJG, drafted this manuscript. All authors revised and approved the final version of the manuscript.

Competing interests

No competing interests to declare.

Funding

This Welsh arm of the BEACHES study was funded by the UK Medical Research Council (MRC) grant number MR/T039329/1. The Australian arm of the BEACHES study was funded by the Australian National Health and Medical Research Council - UKRI-NHMRC Built Environment Prevention Research Scheme (GNT1192764). This research is supported (partially) by the Australian Government through the Australian Research Council's Centre of Excellence for Children and Families over the Life Course (Project ID CE200100025). HC is supported by an Australian National Heart Foundation Future Leader Fellowship (102549). The ORIGINS Project has received core funding support from the Telethon Perth Children's Hospital Research Fund, Joondalup Health Campus, the Paul Ramsay Foundation and the Commonwealth Government of Australia through the Channel 7 Telethon Trust. Substantial in-kind support has been provided by Telethon Kids Institute and Joondalup Health Campus. The Millennium Cohort Study is funded by grants to the Centre for Longitudinal Studies at the Institute of Education from the Economic and Social Research Council and a consortium of government department.

For peer review only - http://bmjopen.bmj.com/site/about/guidelines.xhtml

For peer review only

References

1 Bentham J, Di Cesare M, Bilano V, et al. Worldwide trends in body-mass index, underweight, overweight, and obesity from 1975 to 2016: a pooled analysis of 2416 population-based measurement studies in 128·9 million children, adolescents, and adults. *Lancet*. 2017;390(10113).

2 WHO. Report of the Commission on Ending Childhood Obesity: implementation plan Report by the Secretariat [Internet]. 2017. Available from: http://apps.who.int/iris/bitstream/10665/204176/1/9789241510066_eng.pdf?ua=1

3 Coppinger T, Milton K, Murtagh E, et al. Global Matrix 3.0 physical activity report card for children and youth: a comparison across Europe. *Public Health*. 2020;187:150–6.

4 Christian HE, Bull FC, Middleton NJ, et al. How important is the land use mix measure in understanding walking behaviour? Results from the RESIDE study. *Int J Behav Nutr Phys Act*. 2011;8(1):55. Available from: http://ijbnpa.biomedcentral.com/articles/10.1186/1479-5868-8-55

5 Foster S, Wood L, Christian H, et al. Planning safer suburbs: Do changes in the built environment influence residents' perceptions of crime risk? *Soc Sci Med*. 2013;97:87–94.

6 Christian H, Knuiman M, Divitini M, et al. A Longitudinal Analysis of the Influence of the Neighborhood Environment on Recreational Walking within the Neighborhood: Results from RESIDE. *Environ Health Perspect*. 2017;125(7):077009. Available from: https://ehp.niehs.nih.gov/doi/10.1289/EHP823

7 Müller-Riemenschneider F, Pereira G, Villanueva K, et al. Neighborhood walkability and cardiometabolic risk factors in australian adults: An observational study. *BMC Public Health*. 2013;13(1):755. Available from: http://bmcpublichealth.biomedcentral.com/articles/10.1186/1471-2458-13-755

8 Nobles J, Summerbell C, Brown T, et al. A secondary analysis of the childhood obesity prevention Cochrane Review through a wider determinants of health lens: implications for research funders, researchers, policymakers and practitioners. *Int J Behav Nutr Phys Act*. 2021;18(1). Available from: https://pubmed.ncbi.nlm.nih.gov/33563281/

9 Dixon BN, Ugwoaba UA, Brockmann AN, et al. Associations between the built environment and dietary intake, physical activity, and obesity: A scoping review of reviews. *Obes Rev*. 2021;22(4):e13171. Available from: https://onlinelibrary.wiley.com/doi/10.1111/obr.13171

10 Jia P, Zou Y, Wu Z, et al. Street connectivity, physical activity, and childhood obesity: A systematic review and meta-analysis. *Obes Rev*. 2021;22(S1):e12943. Available from: https://onlinelibrary.wiley.com/doi/10.1111/obr.12943

11 Volger S, Rigassio Radler D, Rothpletz-Puglia P. Early childhood obesity prevention efforts through a life course health development perspective: A scoping review. Ells L, editor. *PLoS One*. 2018;13(12):e0209787. Available from: https://dx.plos.org/10.1371/journal.pone.0209787

12 Wu S, Ding Y, Wu F, et al. Socio-economic position as an intervention against overweight and obesity in children: A systematic review and meta-analysis. *Sci Rep*. 2015;5. Available from: https://pubmed.ncbi.nlm.nih.gov/26112253/

13 Lobstein T, Jackson-Leach R, Moodie ML, et al. Child and adolescent obesity: Part of a bigger

For peer review only - http://bmjopen.bmj.com/site/about/guidelines.xhtml

picture [Internet]. Vol. 385, The Lancet. Lancet Publishing Group; 2015. p. 2510–20. Available from: /pmc/articles/PMC4594797/

14 Blüher M. Obesity: global epidemiology and pathogenesis [Internet]. Vol. 15, Nature Reviews Endocrinology. Nature Publishing Group; 2019. p. 288–98. Available from: https://pubmed.ncbi.nlm.nih.gov/30814686/

15 Coghlan AT, Preskill H, Tzavaras Catsambas T. An overview of appreciative inquiry in evaluation. *New Dir Eval*. 2003;2003(100):5–22. Available from: https://onlinelibrary.wiley.com/doi/full/10.1002/ev.96

16 Cooperrider DL, Whitney DK. Appreciative inquiry : a positive revolution in change [Internet]. . ReadHowYouWant. com; 111 p. Available from: https://books.google.com/books/about/Appreciative_Inquiry.html?id=bJBgktz2Oa8C

17 Public Health Wales. Creating healthier places and spaces for our present and future generations [Internet]. 2018. Available from: www.nationalarchives.gov.uk/doc/open-government-licence/version/3/

18 Klepac Pogrmilovic B, O'Sullivan G, Milton K, et al. The development of the Comprehensive Analysis of Policy on Physical Activity (CAPPA) framework. *Int J Behav Nutr Phys Act*. 2019;16(1):1–15. Available from: https://ijbnpa.biomedcentral.com/articles/10.1186/s12966-019-0822-5

19 Mizen A, Song J, Fry R, et al. Longitudinal access and exposure to green-blue spaces and individual-level mental health and well-being: Protocol for a longitudinal, population-wide record-linked natural experiment. *BMJ Open*. 2019;9(4). Available from: https://pubmed.ncbi.nlm.nih.gov/31005938/

20 Mavoa S, Bagheri N, Koohsari MJ, et al. How Do Neighbourhood Definitions Influence the Associations between Built Environment and Physical Activity? *Int J Environ Res Public Health*. 2019;16(9):1501. Available from: https://www.mdpi.com/1660-4601/16/9/1501/htm

21 Noardo F, Lingua A, Aicardi I, et al. Cartographic data harmonisation for a cross-border project development. *Appl Geomatics*. 2016;8(3):133–50.

22 Fry R, Orford S, Rodgers S, et al. A best practice framework to measure spatial variation in alcohol availability. *Environ Plan B Urban Anal City Sci*. 2020;47(3):381–99. Available from: http://journals.sagepub.com/doi/10.1177/2399808318773761

23 Hyatt M, Rodgers SE, Paranjothy S, et al. The wales electronic cohort for children (WECC) study. *Arch Dis Child - Fetal Neonatal Ed*. 2011;96(Suppl 1):Fa18–Fa18. Available from: https://fn.bmj.com/content/96/Suppl_1/Fa18.1

24 Connelly R, Platt L. Cohort profile: UK Millennium Cohort Study (MCS). *Int J Epidemiol*. 2014;43(6):1719–25.

25 Fitzsimons E BD. Obesity prevalence and its inequality from childhood to adolescence [Internet]. Available from: https://cls.ucl.ac.uk/wp-content/uploads/2020/12/CLS-briefing-paper-Obesity-prevalence-MCS-age-17-initial-findings.pdf

26 Griffiths LJ, Cortina-Borja M, Sera F, et al. How active are our children? Findings from the Millennium Cohort Study. *BMJ Open*. 2013;3(8):e002893. Available from: https://bmjopen.bmj.com/content/3/8/e002893

27 Fluharty M, Villadsen A, Kandola A, et al. Physical activity across age and study: a guide to data in six CLOSER studies Resource report. 2020.

For peer review only - http://bmjopen.bmj.com/site/about/guidelines.xhtml

28 Maddock J, O'neill D, Robinson S, et al. A guide to the dietary data in eight CLOSER studies Resource report. 2020.

29 Christian H, Rosenberg M, Trost S, et al. A snapshot of the PLAYCE project: Findings from the Western Australian PLAY Spaces and Environments for Children's Physical Activity Study. Supportive Childcare Environments for Physical Activity in the Early Years. Perth, Western Australia: The Universit. 2018 Dec.

30 Christian H, Maitland C, Enkel S, et al. Influence of the day care, home and neighbourhood environment on young children's physical activity and health: protocol for the PLAYCE observational study. *BMJ Open*. 2016;6(12):14058. Available from: http://dx.doi.org/10.1136/bmjopen-2016-014058

31 Pate RR, O'Neill JR, Mitchell J. Measurement of physical activity in preschool children. *Med Sci Sports Exerc*. 2010;42(3):508–12.

32 Hinkley T, Salmon J, Okely AD, et al. The HAPPY study: development and reliability of a parent survey to assess correlates of preschool children's physical activity. *J Sci Med Sport*. 2012;15(5):407–17.

33 Burdette HL, Whitaker RC, Daniels SR. Parental report of outdoor playtime as a measure of physical activity in preschool-aged children. *Arch Pediatr Adolesc Med*. 2004;158(4):353–7.

34 Hendrie GA, Viner Smith E, Golley RK. The reliability and relative validity of a diet index score for 4-11-year-old children derived from a parent-reported short food survey. *Public Health Nutr*. 2014;17(7):1486–97. Available from: https://pubmed.ncbi.nlm.nih.gov/23830074/

35 Silva DT, Hagemann E, Davis JA, et al. Introducing the ORIGINS project: a community-based interventional birth cohort. *Rev Environ Health*. 2020;35(3):281–93. Available from: https://www.degruyter.com/document/doi/10.1515/reveh-2020-0057/html

36 Collins CE, Burrows TL, Truby H, et al. Comparison of energy intake in toddlers assessed by food frequency questionnaire and total energy expenditure measured by the doubly labeled water method. *J Acad Nutr Diet*. 2013;113(3):459–63. Available from: https://pubmed.ncbi.nlm.nih.gov/23317500/

37 Soloff C, Lawrence D, Misson S, et al. The Longitudinal Study of Australian Children: an Australian Government initiative - Wave 1 weighting and non-response. 2006.

38 Yu M, Baxter J. 5. Australian children's screen time and participation in extracurricular activities - LSAC Annual Statistical Report 2015.

39 Lyons RA, Jones KH, John G, et al. The SAIL databank: Linking multiple health and social care datasets. *BMC Med Inform Decis Mak*. 2009;9(1):3. Available from: https://bmcmedinformdecismak.biomedcentral.com/articles/10.1186/1472-6947-9-3

40 Ford D V., Jones KH, Verplancke JP, et al. The SAIL Databank: Building a national architecture for e-health research and evaluation. *BMC Health Serv Res*. 2009;9(1):157.

41 Johnson RD, Griffiths LJ, Hollinghurst JP, et al. Deriving household composition using population-scale electronic health record data—A reproducible methodology. *PLoS One*. 2021;16(3):e0248195. Available from: https://journals.plos.org/plosone/article?id=10.1371/journal.pone.0248195

42 Hooper P, Foster S, Bull F, et al. Living liveable? RESIDE's evaluation of the "Liveable Neighborhoods" planning policy on the health supportive behaviors and wellbeing of residents in Perth, Western Australia. *SSM - Popul Heal*. 2020;10:100538.

For peer review only - http://bmjopen.bmj.com/site/about/guidelines.xhtml

43 Davies HT, Powell AE, Nutley SM. Mobilising knowledge to improve UK health care: learning from other countries and other sectors – a multimethod mapping study. *Heal Serv Deliv Res*. 2015;3(27):1–190. Available from: https://www.ncbi.nlm.nih.gov/books/NBK299400/

Figure Legend

Figure 1 - BEACHES project workflow.

Additional Information

Acknowledgements
This study makes use of anonymised data held in the Secure Anonymised Information Linkage (SAIL) Databank. We would like to acknowledge all the data providers who make anonymised data available for research. Partners for the Australian arm include the Western Australian (WA) Department of Local Government, Sport and Cultural Industries, WA Department of Health, WA Department of Transport, WA Local Government Association, Australian Childcare Alliance, Nature Play Australia, Heart Foundation, Cancer Council WA, Goodstart Early Learning, and Hames Sharley. The ORIGINS Project is only possible because of the commitment of the families in ORIGINS. We are grateful to all the participants, health professionals and researchers who support the project. We would also like to acknowledge and thank the following teams and individuals who have made The ORIGINS Project possible: The ORIGINS Project team; CEO Dr Kempton Cowan, executive staff and obstetric, neonatal and paediatric teams, Joondalup Health Campus (JHC); Director Professor Jonathan Carapetis and executive staff, Telethon Kids Institute; Mayor Tracey Roberts, City of Wanneroo; Mayor Albert Jacobs, City of Joondalup; Professor Fiona Stanley, patron of ORIGINS; members of ORIGINS Community Reference and Participant Reference Groups; Research Interest Groups and the ORIGINS Scientific Committee. BEACHES is a sub-project of The ORIGINS Project. This unique long-term study, a collaboration between Telethon Kids Institute and Joondalup Health Campus, is one of the most comprehensive studies of pregnant women and their families in Australia to date, recruiting 10,000 families over a decade from the Joondalup and Wanneroo communities of Western Australia.

Disclaimer
The views expressed in this paper are those of the authors and not necessarily those of the funding bodies, the NHS, The NIHR, or the Department of Health and Social Care.

Acronyms

BMI	Body Mass Index
CMPW	Child Measurement Programme for Wales
GIS	Geographic Information Systems
LSAC	Longitudinal Study of Australian Children
MCS	Millennium Cohort Study
NCD	Non-Communicable Disease

For peer review only - http://bmjopen.bmj.com/site/about/guidelines.xhtml

OS	Ordnance Survey
OSM	Open Street Map
PLAYCE	PLAY Spaces & Environments for Children's Physical Activity
RALF	Residential Anonymised Linking Field
SAIL	Secure Anonymised Information Linkage
WA	Western Australia
WECC	Wales Electronic Cohort for Children (WECC)
UK	United Kingdom

For peer review only

For peer review only - http://bmjopen.bmj.com/site/about/guidelines.xhtml

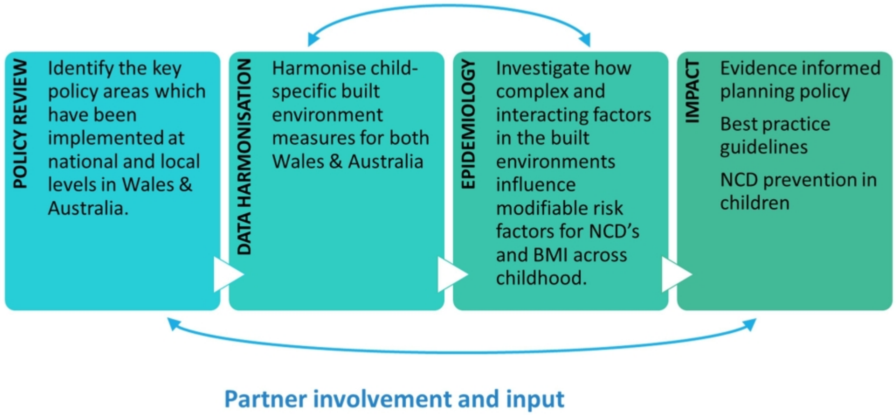

BEACHES project workflow

297x209mm (300 x 300 DPI)

BMJ Open

Built Environment and Child Health in Wales and Australia (BEACHES): a study protocol

Journal:	*BMJ Open*
Manuscript ID	bmjopen-2022-061978.R1
Article Type:	Protocol
Date Submitted by the Author:	05-Jul-2022
Complete List of Authors:	Pedrick-Case, Rebecca; Swansea University Medical School, Population Data Science<br>Bailey, Rowena; Swansea University Medical School, Population Data Science<br>Beck, Ben; Monash University, School of Public Health and Preventive Medicine<br>Beesley, Bridget; The University of Western Australia, UWA School of Agriculture and Environment<br>Boruff, Bryan; The University of Western Australia, UWA School of Agriculture and Environment<br>Brophy, Sinead; Swansea University Medical School, Population Data Science<br>Cross, Donna; The University of Western Australia, Telethon Kids Institute; The University of Western Australia, School of Population and Global Health<br>Dhamrait, Gursimran; The University of Western Australia, Telethon Kids Institute; The University of Western Australia, School of Population and Global Health<br>Duncan, John; University of Western Australia, UWA School of Agriculture and Environment<br>Gething, Peter; The University of Western Australia, Telethon Kids Institute; Curtin University, Faculty of Health Sciences<br>Johnson, Rhodri; Swansea University Medical School, Population Data Science<br>Lyons, Ronan; Swansea University Medical School, Population Data Science<br>Mizen, Amy; Swansea University Medical School, Population Data Science<br>Murray, Kevin; University of Western Australia, School of Population and Global Health<br>Pouliou, Theodora; Swansea University Medical School, Population Data Science<br>Rafferty, James; Swansea University Medical School, Population Data Science<br>Rosenberg, Michael; University of Western Australia, School of Human Sciences<br>Schipperijn, Jasper; University of Souther Denmark, Department of Sports Science and Clinical Biomechanics<br>Thompson, Daniel; Swansea University Medical School, Population Data Science<br>Trost, Stewart; The University of Queensland, School of Human

	Movement and Nutrition Sciences<br>Watkins, Alan; Swansea University Medical School<br>Stratton, Gareth; Swansea University, Research Centre in Applied Sports, Technology, Exercise and Medicine<br>Fry, Richard; Swansea University Medical School, Population Data Science<br>Christian, Hayley; The University of Western Australia, Telethon Kids Institute; The University of Western Australia, School of Population and Global Health<br>Griffiths, Lucy ; Swansea University, Swansea University Medical School
**Primary Subject Heading**:	Epidemiology
Secondary Subject Heading:	Epidemiology
Keywords:	EPIDEMIOLOGY, PAEDIATRICS, PREVENTIVE MEDICINE, PUBLIC HEALTH

Built Environment and Child Health in Wales and Australia (BEACHES): a study protocol

Rebecca Pedrick-Case[1], Rowena Bailey[1], Ben Beck[2], Bridget Beesley[3], Bryan Boruff[3], Sinead Brophy[1], Donna Cross [4,5], Gursimran Dhamrait [4,5], John Duncan[3], Peter Gething[4,6], Rhodri D Johnson[1], Ronan A Lyons[1], Amy Mizen[1], Kevin Murray[5], Theodora Pouliou[1], James Rafferty[1] Michael Rosenberg[7], Jasper Schipperijn[8], Daniel A Thompson[1], Stewart Trost[9], Alan Watkins[1], Gareth Stratton[10, #], Richard Fry[1,#], Hayley Christian [4,5,#], Lucy J Griffiths[1,#,*]

[1] Population Data Science, Swansea University Medical School, Swansea, UK.

[2] School of Public Health and Preventive Medicine, Monash University, Melbourne, Australia.

[3] UWA School of Agriculture and Environment, University of Western Australia, Perth, Australia.

[4] Telethon Kids Institute, University of Western Australia, Perth, Australia.

[5] School of Population and Global Health, University of Western Australia, Perth, Australia.

[6] Faculty of Health Sciences, Curtin University, Perth, Australia.

[7] School of Human Sciences, University of Western Australia, Perth, Australia.

[8] Department of Sports Science and Clinical Biomechanics, University of Southern Denmark, Odense, Denmark.

[9] School of Human Movement and Nutrition Sciences, University of Queensland, Brisbane, Australia.

[10] Research Centre in Applied Sports, Technology, Exercise and Medicine (A-STEM), Swansea University, Swansea, UK.

* Corresponding author: Dr Lucy Griffiths, Swansea University Medical School, Swansea, University, Swansea, UK; lucy.griffiths@swansea.ac.uk

# Joint senior authors

ORCID IDs

For peer review only - http://bmjopen.bmj.com/site/about/guidelines.xhtml

Rebecca Pedrick-Case: 0000-0003-2307-1808

Rowena Bailey: 0000-0003-2409-2045

Ben Beck: 0000-0003-3262-5956

Bridget Beesley: 0000-0003-0432-3766

Bryan Boruff: 0000-0001-6693-0671

Sinead Brophy: 0000-0001-7417-2858

Donna Cross: 0000-0001-6217-5058

Gursimran K Dhamrait: 0000-0002-5191-211X

John Duncan: 0000-0001-9752-1002

Peter Gething: 0000-0001-6759-5449

Rhodri D Johnson: 0000-0001-9636-0753

Ronan A Lyons: 0000-0001-5225-000X

Amy Mizen: 0000-0001-7516-6767

Kevin Murray: 0000-0002-8856-6046

Theodora Pouliou: 0000-0002-1162-1174

James Rafferty: 0000-0002-1667-7265

Michael Rosenberg: 0000-0002-9950-5775

Jasper Schipperijn: 0000-0002-6558-7610

Daniel A Thompson: 0000-0002-1769-2870

Stewart Trost: 0000-0001-9587-3944

Alan Watkins: 0000-0003-3804-1943

Rich Fry: 0000-0002-7968-6679

Gareth Stratton: 0000-0001-5618-0803

Hayley Christian: 0000-0001-8486-5746

Lucy J Griffiths: 0000-0001-9230-624X

For peer review only

Word Count: 3568/4,000 words

Keywords: Non-communicable disease, childhood obesity, built environment, linked data, cohort studies, ORIGINS Project, PLAYCE Cohort Study, Longitudinal Study of Australian Children, Millennium Cohort Study, SAIL Databank.

For peer review only - http://bmjopen.bmj.com/site/about/guidelines.xhtml

Abstract

Introduction: Childhood obesity and physical inactivity are two of the most significant modifiable risk factors for the prevention of non-communicable diseases (NCDs). Yet, a third of children in Wales and Australia are overweight or obese, and only 20% of UK and Australian children are sufficiently active. The purpose of this study is to identify and understand how complex and interacting factors in the built environment influence modifiable risk factors for NCDs across childhood.

Methods & Analysis: This is an observational study using data from five established cohorts from Wales and Australia: 1) Wales Electronic Cohort for Children (WECC); 2) Millennium Cohort Study (MCS); 3) PLAY Spaces & Environments for Children's Physical Activity (PLAYCE) study; 4) the ORIGINS Project; and 5) The Growing Up in Australia: the Longitudinal Study of Australian Children (LSAC). The study will incorporate a comprehensive suite of longitudinal quantitative data (surveys, anthropometry, accelerometery, and Geographic Information Systems (GIS) data) to understand how the built environment influences children's modifiable risk factors for NCDs (Body Mass Index (BMI), physical activity, sedentary behaviour, and diet).

Ethics & Dissemination: This study has received the following approvals: The University of Western Australia Human Research Ethics Committee (2020/ET000353), Ramsay Human Research Ethics Committee (under review), and Swansea University Information Governance Review Panel approval (Project ID: 1001). Findings will be reported to the following: 1) funding bodies, research institutes and hospitals supporting the BEACHES project; 2) parents and children; 3) school management teams; 4) existing and new industry partner networks; 5) federal, state, and local governments to inform policy; as well as 6) presented at local, national, and international conferences; and 7) disseminated by peer-review publications.

For peer review only

For peer review only - http://bmjopen.bmj.com/site/about/guidelines.xhtml

Article Summary

Strengths and limitations of this study

- The BEACHES project uses large representative samples of children from five Wales and Australian cohort studies.
- Standardised built environment measures will be applied across Wales and Australia and linked to the cohort study data at the individual-level.
- The contrasting timepoints, climates, geographies, and policy approaches in Wales and Australia will provide stronger evidence of the causal pathways between the built environment and child health.
- The use of existing cohort datasets can limit the use of consistent health outcomes across studies.
- Analyses using routinely recorded data (Wales) may omit some unknown confounders, thereby introducing a moderate level of bias due to confounding.

Introduction

Childhood obesity and physical inactivity are two of the most significant modifiable risk factors for non-communicable disease (NCD) prevention in children.[1] The 2017 Commission on Ending Childhood Obesity emphasised that the prevention of modifiable risk factors for NCDs should start as early as possible.[2] Yet only 20% of UK and Australian children are sufficiently active, and over 60% engage in excessive sedentary time, with a third overweight or obese.[3]

The built environment in which we live is integral to human health. Research has shown that residing in 'liveable' neighbourhoods characterised by: good access to shops, services, quality parks, connected streets to facilitate walking, sufficient residential densities to support public transport services and local businesses, minimal crime and traffic, and social connectedness opportunities are associated with improved health outcomes.[4–9]. Despite the increasing evidence of the association between the built environment and physical activity, there is still a paucity of longitudinal research examining the role of the built environment in promoting child health.[10,11] Critical evidence gaps for young people include: 1) causal evidence of the overall impact the built environment has on children's modifiable risk factors for NCDs (i.e., physical inactivity, sedentariness, and poor diet);[11,12] 2) the specific effect

For peer review only - http://bmjopen.bmj.com/site/about/guidelines.xhtml

For peer review only

of individual, familial, and combinations of built environment attributes; 3) how these effects vary across different ages of children;[13] 4) how these effects are moderated by socioeconomic status;[14] 5) the mediating role of physical activity and healthy eating on the relationship between the built environment and child obesity;[15] and 6) how the influence of the built environment on children's modifiable risk factors for NCDs varies by different geographical locations.[16] These evidence gaps hinder the formulation of specific, actionable policies to improve the built environment for child health.

The Built Environment and Child Health in Wales and Australia (BEACHES) project is a collaboration between academic institutions in Wales and Australia to identify and understand how complex and interacting factors in the built environment influence modifiable risk factors for NCDs across childhood. This project aims to address identified evidence gaps by using Wales and Australian longitudinal population linked and cohort data to identify and understand how complex and interacting factors in the built environment influence modifiable risk factors for NCDs (physical inactivity, sedentariness, poor diet) across childhood. Five large Welsh and Australian cohort studies, with detailed anthropometric, physical (in)-activity, diet, and contextual data for more than one million children will be used. We will use the highest quality available spatial data and geospatial techniques to construct an internationally standardised set of metrics, such as walkability, that characterise the built environments each child has inhabited during childhood. A statistical modelling framework will be used to quantify the influence that different built environment characteristics have on children's BMI, and the respective roles of physical activity, sedentary behaviour, and diet in this relationship.

Objectives

The purpose of this study is to identify and understand how complex and interacting factors in the built environment influence modifiable risk factors for NCDs across childhood. The objectives are to:

1. Develop a comprehensive Geographic Information Systems (GIS) model of child-specific built environment characteristics using standardised methods for Wales and Australia,
2. Link standardised GIS models of the built environment to e-cohort and standard cohort data for children in Wales and Australia,
3. Determine the direct and indirect (and mediating) relationships between the built environment and: a) children's modifiable risk factors for NCDs (physical activity, sedentary time, and diet), and b) obesity,

For peer review only - http://bmjopen.bmj.com/site/about/guidelines.xhtml

4. Identify how relationships between the built environment and these NCD risk factors vary by children's socio-economic position and geographic location (across and within Wales and Australia), and
5. Produce evidence which policymakers and other stakeholders can use to modify the built environment to enable physical activity and reduce childhood obesity.

Methods and analysis

This multi-site study (funding period: 2020-2023) will use population-level cohort data (including but not limited to surveys, anthropometry, accelerometry, GIS data, and biological samples) to address these five objectives (Figure 1). We will review the policy landscape and literature on the key built environment and child health-related policies at national and local levels in Wales and Australia. Based on the findings, we will curate and harmonise environmental data that will be used to develop high resolution spatial models of the built environment. These built environment measures will be linked with cohort and population-level health data (Wales) to identify and understand how complex and interacting factors in the built environment influence modifiable risk factors for NCDs and BMI across childhood using a series of covariate-adjusted, multi-level regression models. Finally, we will communicate to policymakers and planners which modifiable aspects of the built environment may contribute to a reduction in risk factors for NCDs and BMI across childhood.

INSERT Figure 1

Policy & Literature Review

We will actively engage with stakeholders and carry out a policy landscape review (for the duration of the cohort studies) to examine key built environment and child health-related policies at national and local levels in Wales and Australia. Findings will provide the overall context for policy gaps and landscapes, and the co-design of research to address these gaps, develop best practice guidelines and overall, reduce NCDs in children.

Our stakeholders include health service commissioners, government departments of planning, transport and health, planning officials, urban planners, building industry, developers and key advocacy non-governmental organisations, as well as third sector and voluntary agencies.

For peer review only - http://bmjopen.bmj.com/site/about/guidelines.xhtml

In Wales, we will engage with our stakeholders at three time points throughout the project. These include, for example, Public Health Wales, Active Healthy Kids Wales and Play Wales. We will use appropriate frameworks (e.g. 'appreciative inquiry') [17,18] to inform and interpret our quantitative research findings into stakeholder engagement activities. We will map out policies and key legislation across Wales using the six priority areas of the built and natural environment that Public Health Wales has published in the 'Creating healthier places and spaces for our present and future generations 'report.[19]

In Australia, we plan to meet our government and non-government partners quarterly each year for the length of the grant. These include the Western Australian (WA) Department of Local Government, Sport and Cultural Industries, WA Department of Health, WA Department of Transport, WA Local Government Association, Australian Childcare Alliance, Nature Play Australia, Heart Foundation, The PLAY Spaces and Environments for Children's Physical Activity (PLAYCE) partners, Cancer Council WA, Goodstart Early Learning, and Hames Sharley. With our partners, we will conduct a policy analysis to investigate how Western Australian and national policies address the health of children through the built environment's influence on obesity and the modifiable risk factors for obesity, physical activity, sedentary behaviour, and diet. Policy analysis is crucial to achieving reforms in health promotion by raising awareness of current policy gaps and opportunities and demonstrating successful policy related actions being taken across the system. The Comprehensive Analysis of Policy on Physical Activity (CAPPA) framework will be used to guide the analysis.[20]

Built Environment Measures

GIS-derived built environment measures will be calculated at the residential address level for Wales and Australia. Table 1 outlines the standardised built environment measures that will be created for all Wales and Australian datasets. We will use rich vector spatial datasets which define land utilisation (e.g. building footprint and height, cadastre, road centreline and reserve, and points of interest), planning data (e.g. active travel routes, open space assessments, fast food outlets) and Earth Observation data (e.g. Landsat, Sentinel, Aerial Photography). We have developed a broad range of longitudinal data and methods which characterise the built environment of the two study areas (Table 2). These include neighbourhood walkability, garden size/home outdoor area, blue-green space availability,[21] access to services and facilities, and fast-food outlets around the home and school.

In previous studies of built-environment measures for physical activity, how the neighbourhood is defined has greatly influenced the results.[22] For example, access to green and blue spaces or destinations (retail and food outlets) can be based on distance from a child's residence measured as a

For peer review only - http://bmjopen.bmj.com/site/about/guidelines.xhtml

straight-line (Euclidean distance) from a household to a destination or based on movement along a road network, with often very different results. Therefore, the GIS and epidemiological teams will work together closely with a 'behaviour-led approach' to define the neighbourhood sizes most likely to correlate with the behaviour of interest by age group. For example, active travel from home to school.

Harmonisation of Built Environment Measures

As far as reasonably possible, the data, spatial analytic approaches, and built environment measures will be standardised and harmonised across the Welsh and Australian study cohorts to enable the calculation of comparable measures. This will involve using temporally aligned GIS and cohort data (i.e., built-environment metrics generated using spatially referenced data relating to the epidemiological timepoint of interest), age-specific built environment profiles and neighbourhood characterisations. These data will be sourced, where possible, from open data (e.g., OpenStreetMap) but will also be supplemented with existing data holdings from licenced data holdings (e.g., Ordnance Survey Points of Interest) to provide a rich temporal coverage. Where temporal mismatches occur, we will explore methods to impute exposure metrics using the data available, to align with the appropriate time points. Standardisation and harmonisation will increase confidence in the comparability of measures by minimising potential errors from heterogeneity in data collection procedures, software or methodological approaches; and is particularly important in projects between two countries where definitions, symbology, and projection in source cartographic data will vary.[23] Where feasible, we will use open data (e.g., OSM) and open-source software, such as QGIS, SQL, Python or R, to reduce barriers to reproducing this research in other contexts, thus increasing the potential impact. Methods and code will be made available in future publications, and variations in terminology will be mapped out in a typology with definitions. This will help us to identify where broader policy approaches can be taken and where more contextually specific policies will need to be implemented.[24]

For peer review only

For peer review only - http://bmjopen.bmj.com/site/about/guidelines.xhtml

237 **Table 1.** Wales and Australian Standardised Measures of the Built Environment.

Built Environment Feature	Indicative BE Variables	Data Source	
		**Wales**	**Australia**
**Outdoor home environment**	Home garden/yard space & vegetation	Aerial imagery, NDVI/EVI	Aerial imagery, NDVI/EVI, Urban Monitor, Landgate (Cadastre)
**Child-relevant walkability**	Road traffic exposure, dwelling density, residence type, street connectivity, one-way node count, land use mix, 'local living score', public transport stops	OS Master Map, OSM	Landgate, Main Roads Western Australia, Western Australian Public Transport Authority, Valuer Generals Office, OSM
**Child-relevant green/blue space**	Public open space type (park, school grounds, natural, club/playgrounds, residual greenspace), park size category (Pocket/small, medium/large, district/regional), park features and attractiveness, vegetation, blue-space (distance to coast)	OS Master Map & Greenspace, OSM	University of Western Australia – Centre for the Built Environment and Health, Landgate
**Child-relevant destinations and features**	Playgroups, childcare centres, before and after school care services, kindergarten/nurseries, primary school, secondary school, other	Local authority data, OSM	Playgroup WA Inc, Western Australian Government Department of Local Government, Sport and Cultural Industries' Department of Education, Department of Transport, Department of Planning, Lands and Heritage, Department of Health
**Socioeconomic status**	Such as household level of income.	WIMD, individual-level SES data in SAIL databank	Socio-Economic Indexes for Areas (SEIFA)
**Household location**	Required to derive other measures	UPRN (OS), linked to RALF within SAIL database.	Property Street Address (PSA), GNAF

238 NDVI: Normalised Difference Vegetation Index; EVI: Enhanced Vegetation Index, OSM: Open Street Map, OS: Ordnance Survey, ONS: Office for National Statistics, SAIL: Secure Anonymised
239 Information Linkage; UPRN: Unique Property Reference Number, RALF: Residential Anonymised Linking Field, GNAF: Geoscape Geocoded National Address File, SES: socio-economic status.

For peer review only - http://bmjopen.bmj.com/site/about/guidelines.xhtml

Data Sources, Linkage and Statistical Analysis (Epidemiology)

Built environment models (see above) will be linked with child cohort data to investigate how complex and interacting factors in the built environments may influence modifiable risk factors for NCD's and BMI across childhood.

Data Sources

Five large Welsh and Australian cohort studies will be used. They include: 1) the Wales Electronic Cohort for Children (WECC); 2) the Millennium Cohort Study (MCS Wales); 3) the PLAY Spaces & Environments for Children's Physical Activity (PLAYCE) study (Australia); 4) the ORIGINS Project (Australia); and 5) The Growing Up in Australia: The Longitudinal Study of Australian Children (LSAC; Table 2). Further details about each of the datasets are provided below. The age range covers both childhood and adolescence, with multiple data collection dates for each cohort study.

Table 2. Overview of Wales and Australian health datasets.

Characteristics	Datasets & Location				
	**WECC (Wales)**	**MCS (Wales)**	**PLAYCE (AU)**	**ORIGINS (AU)**	**LSAC (AU)**
**Age**	0-17	5-14	2-9	1-3	9-17
**Sample size**	1.3 million	1,699-2,181	2,028	2,036	8,416
**No. time points**	Dynamic	4	3	2	8
**Collection years**	1990-ongoing	2006-2015	2015-2022	2018-2022	2008-2018
**BMI**	*				
**Physical Activity**	X				
**Sedentary time**	X				
**Diet**	X				
**Confounders**					
**Geolocation**	Home, school	Home, School	Home, childcare, school	Home	Home

UK: United Kingdom, AU: Australia, WECC: Wales Electronic Cohort for Children, MSC: Millennium Cohort Study, PLAYCE: PLAY Spaces and Environments for Children's Physical Activity, LSAC: The Longitudinal Study of Australian Children. *BMI data is available for approximately *n*=244,000 children from the Child Measurement Programme in Wales.

Wales

<u>The Wales Electronic Cohort for Children (WECC)</u>

WECC is a total population anonymised electronic cohort study of all children born and living in Wales since 1990 (Table 2) (n=1.3 million) which is updated every three months. The WECC includes information for all live births (approximately 35,000 births per year), Welsh resident mother at birth, or a child who becomes a resident in Wales as identified by registering with a GP practice in Wales.

For peer review only - http://bmjopen.bmj.com/site/about/guidelines.xhtml

This will be supplemented with BMI data from the Child Measurement Programme in Wales (measured height and weights at age 5 years) and from population-level health data (general practitioner records, hospital admissions) for those children and young people who have follow-up (post age 5) heights, weights or BMI values in their health records. This dataset also includes linkage to total population education data and a subset of child reported measures (20,000+) including diet, physical activity/physical literacy, wellbeing, child-assessed rating of local area for safety/play. A fuller description of the WECC and its contents can be found in Hyatt et al.[25]

<u>The Millennium Cohort Study (MCS)</u>

The MCS is a multi-disciplinary research project following the lives of around 19,000 young people born across England, Scotland, Wales and Northern Ireland in 2000-02. .[26] The original cohort comprised 18 818 children (72% of those approached) whose parents were first interviewed at home when their child was around aged 9 months. Since then, data have been collected regularly throughout childhood and adolescence. This research aims to use information relating to approximately 2,000 children and young people resident in Wales at ages 5 (2006), 7 (2008), 11 (2012) and 14 years (2015). Children's height, weight and waist circumference were measured using standard protocols. [27] Physical activity was measured using accelerometery at age 7 [28] and, across all age groups, parent-reported measures of sedentary (e.g. parent-report screen time) and physical activity behaviours (e.g. sports club and playing outside),[29,30] as well as dietary factors (e.g. snacking, breakfast consumption) and a range of covariate information relating to socio-economic and demographic factors of the cohort and both resident parents were recorded.

Australia

<u>PLAY Spaces & Environments for Children's Physical Activity (PLAYCE – Western Australia)</u>

The PLAY Spaces & Environments for Children's Physical Activity (PLAYCE) is an ongoing cohort study (2015-current), investigating the influence of the childcare-, home-, and neighbourhood- built environments (parent perceptions and objectively measured) on children's physical activity, sedentary behaviour, diet and weight status.[31] Baseline data were collected for 2,028 children, aged 2-5 years, recruited from 118 childcare centres (response rate 46%) in metropolitan Perth, Western Australia. Childcare centres were recruited evenly across socio-economic statuses and size (small and large). Two additional data time points were collected once children commenced formal schooling, at 5-7 years (2019-20) and 7-9 years (2020-21). The study design enables modifiable risk factors to be examined as children transition between different built environment settings: home, childcare; and full-time school. Full details of the PLAYCE study protocol have been published.[32]

For peer review only

Children's height, weight and waist circumference were measured by the research team using standard protocols. Physical activity was measured using accelerometery, and parent-report structured and unstructured physical activity including outdoor play.[32–35] Sedentary behaviour was measured using accelerometery (time spent sedentary) and parent-report screen time.[32–34] Diet was assessed using a parent-report short food frequency questionnaire.[36]

The ORIGINS Project (Western Australia)

ORIGINS is a collaborative initiative between the Telethon Kids Institute and Joondalup Health Campus. It is a birth cohort of 10,000 families enrolled during pregnancy and followed children from birth to age five.[37] ORIGINS is fully integrated into clinical and diagnostic services. It aims to improve understanding of the early antecedent pathways that mediate NCDs development through the study of early environments, maternal and paternal physical health, and genetics. ORIGINS overarching goal is to reduce the rising epidemic of NCDs by providing children with 'a healthy start to life.' In 2021, the cohort will include data on 2,036 children collected at 12 and 36 months.

Children's height, weight and waist circumference were measured by trained staff. Physical activity was measured using parent-report structured and unstructured physical activity as well as outdoor play.[35] Sedentary behaviour was measured via parent-report screen time. Parents reported their child's diet using the Australian Eating Survey; a validated semi-quantitative food frequency questionnaire.[38]

Longitudinal Study of Australian Children (LSAC - Australia)

Growing Up in Australia: The Longitudinal Study of Australian Children (LSAC) is an ongoing prospective study that used a two cohort cross-sequential design and in 2004 recruited 5,107 children to the baby cohort (~ 9 months) and 4,983 children to the kindergarten cohort (~4.8 years).[39] The sampling method provided a representative sample of Australian children. Data were collected every two years for both cohorts. Data from children aged 9-17 years will be included. Full details of the LSAC study are published elsewhere.[39]

Children's height, weight, body composition, and waist circumference were measured by parents. Physical activity was measured using parent-report time-use diaries which included structured and unstructured physical activity. Sedentary behaviour was gathered through parent reports and the children's time use diary for three types of screen-based activities (television viewing, computer use and electronic games).[40] Each parents reported their child's diet via food diaries. In the Child Health CheckPoint, children completed a questionnaire regarding their usual intake of various foods including fruit and vegetable consumption, water intake, and breakfast consumption.

For peer review only - http://bmjopen.bmj.com/site/about/guidelines.xhtml

Data Linkage

Wales

Routinely collected health data and cohort study data from studies such as the Millennium Cohort Study, are available via the Secure Anonymised Information Linkage (SAIL) databank.[41,42] This is a unique resource of linked longitudinal health, socio-economic and environmental data relating to the people of Wales. One of the core linkage mechanisms is the Residential Anonymised Linking Field (RALF).[43] The benefits of the RALF system are three-fold. First, it allows the GIS modelling of population-level socio-environmental measures for approximately 1.4 million households in Wales at ±1m spatial resolution. Second, it allows the grouping of individuals into household units in SAIL; thus, reducing ecological fallacies and the impacts of the modifiable areal unit problem. Thirdly, it allows house moves to be identified and hence length of exposure to the built environment to be examined.

Australia

Home addresses will be geocoded for all three Australian cohorts (ORIGINS, PLAYCE and LSAC) to enable the creation of GIS measures of the natural and built environment by the Australian team.[32,44] PLAYCE and ORIGINS cohort data will be linked to built environment measures using a unique participant identification number. Where possible, change in addresses over time will be examined for these cohorts. LSAC data linkage will involve the Australian Bureau of Statistics, the Australian Institute of Family Studies' Data Linkage and Integrating Authority, the Australian Institute of Family Studies' Data Management team, National Centre for Longitudinal Data.

Statistical analysis

A series of covariate-adjusted multi-level regression models will be fitted to explore the impact of different aspects of the built environment on BMI/NCD risk factors, to quantify the effect of each associative relationship using a common outcome variable. A multi-level model structure will be used to adjust for inherent autocorrelation that exists within groups of individuals. Covariates will include, for example, child age, sex, socio-economic status (e.g., parent education, neighbourhood level disadvantage), and family composition. We will also examine how these relationships vary by different population groups (e.g., young children, school age children and adolescents). This series of analyses will enable a comparison and prioritisation of the built environment factors most strongly associated with BMI and quantify the magnitude of variation in effect size across various built environment features. We will then standardise these results to rank characteristics of the built environment which may contribute to better health, to assist prioritisation of public services and resources.

For peer review only - http://bmjopen.bmj.com/site/about/guidelines.xhtml

The direct, indirect, and total effects of the built environment on children's BMI and other NCD risk factors will be estimated using well established multi-level mediation modelling. We will also determine if differences in these factors between advantaged and disadvantaged neighbourhoods can be explained in part by differences in built environment attributes (e.g., better or poorer access to green space). The mediation effect of physical activity and the moderation effect of neighbourhood socio-economic disadvantage on the relationship between the built environment and BMI will initially be tested as separate models. A moderated path analysis using all longitudinal data will combine moderation and mediation, and identify direct, indirect, and total mediation effects, and show how these effects vary across the levels of the moderator. All analyses will adjust for baseline and, where appropriate, temporal confounding. Special attention will also be paid to potential confounders of the outcome (e.g. BMI) and the mediating variable (e.g. physical activity) being examined. The role of age, sex, socio-economic status and urban/rural locality will be examined.

Statistical analyses will be conducted separately for Welsh and Australian datasets. A set of built environment measures will be standardised and linked to the five cohort datasets. Data governance prohibits pooling of datasets; however, findings from both countries' analysis of their respective linked health cohort and built environment datasets will be compared to determine differences between different age groups of children in Wales and Australia.

Formal statistical analysis plans will be drafted in advance of the analyses and agreed with our Expert Advisory Group. These will specify our modelling strategies, rules on dealing with missing data, and reporting conventions, including the provision of CONSORT diagrams.

Patient and Public Involvement

Development of the research questions and outcome measures were informed by ongoing engagement with stakeholders who have an interest in child health, such as Public Health Wales, and the PLAYCE Community Reference Group (Western Australia). This study uses anonymised routinely collected and cohort data, so it was not possible to directly consult the individuals included in the study.

Results will be disseminated by engaging children directly such as via a short video that can be shown in schools and online, science outreach workshops, and existing child-health networks to translate the

For peer review only - http://bmjopen.bmj.com/site/about/guidelines.xhtml

findings into useful advice for children, their families, and policy makers. Please refer to 'Ethics & Dissemination' below further information.

Ethics & Dissemination

All data will be anonymised and, in Wales, linked within the privacy protecting SAIL Databank. We will be using anonymised data and therefore we are exempt from National Research Ethics Committee (NREC). An Information Governance Review Panel (IGRP) [Project ID: 1001] provided approval to link these data. The PLAYCE study has existing Human Research Ethics Approval from The University of Western Australia (UWA: #RA/4/1/7417). An application will be submitted to the Ramsay Human Research Ethics Committee Western Australia for the ORIGINS and built environment analyses and to the UWA Human Research Ethics Committee for the LSAC and built environment secondary analyses. All Australian research will abide by the NHMRC's National Statement on Ethical Conduct in Human Research (2008) and is considered negligible risk.

Our dissemination and impact generation strategies will be informed by research on knowledge mobilization models; [45] to impact at a variety of scales (including national and local government and across multiple sectors) and will not be confined to a Welsh-Australian context. Throughout, we will involve stakeholders and policy makers in the research process from inception to translation, seeking their feedback on our process, progress, and findings. We will disseminate findings through community engagement (e.g., at public events for families or to school management teams), and through existing and new industry partner networks. An information sheet will be prepared in plain English with infographics, and a video explaining the project and its findings will be made available online and advertised through social media. Findings from the study will be published in a comprehensive report and an infographic summary. We will notify all stakeholders and promote publication through international networks established during this research. We will hold seminars and workshops to report our findings to stakeholders and the public.

Discussion

The purpose of this study is to identify and understand how complex and interacting factors in the built environment influence modifiable risk factors for NCDs across childhood. Harmonising both child

For peer review only - http://bmjopen.bmj.com/site/about/guidelines.xhtml

and built environment indicators across five large-scale studies enables analysis of an effectively larger sample size, spans a broader set of age-ranges, and leverages greater heterogeneity in built environments. By harmonising analyses from Welsh and Australian settings, the BEACHES project provides a unique opportunity to identify impacts of the built environment that are common across settings and where place-specific, physical, cultural, and policy environments mediate these effects.

We aim to communicate to policy and planners which modifiable aspects of the built environment result in the most significant reductions in the risk factors for NCDs across childhood. This will enable action at both a national and international scale and, in turn, will contribute to population-level reductions in NCDs. This is an essential component of our work to able to bring about large-scale improvement in the built environment to reduce NCDs beginning in early childhood. Furthermore, the outputs of this research could be used to inform future research by aggregating evidence of individual associations in a single dynamic model.

Contributorship statement

HC, GS, LG, and RF are the Principal Investigators who conceptualised the overall study, established research collaborations and led the project implementation. Co-Investigators who contributed to obtaining the research funding include BB (Beck), BB (Boruff), SB, DC, PG, RAL, AM, KM, MR, JS, ST, AW. These Principle and Co-Investigators, in additional other team members (RP-C, RB, BB (Beesley), JD, RDJ, JR, DAT) are responsible for the development of Geographic Information Systems measures and associated data analysis. RB, KM, AW, RDJ, JR, HC and LJG are responsible for the design and implementation of the statistical analyses. GS, TP, HC, GD are responsible for the policy reviews and stakeholder engagement. RP-C, GD, RF, HC, and LJG, drafted this manuscript. All authors revised and approved the final version of the manuscript.

Competing interests

No competing interests to declare.

For peer review only - http://bmjopen.bmj.com/site/about/guidelines.xhtml

For peer review only

Funding

This Welsh arm of the BEACHES study was funded by the UK Medical Research Council (MRC) grant number MR/T039329/1. This work will also be supported by the Administrative Data Research (ADR) Wales, part of the ADR UK investment, which unites research expertise from Swansea University Medical School and WISERD (Wales Institute of Social and Economic Research and Data) at Cardiff University with analysts from Welsh Government. ADR UK is funded by the Economic and Social Research Council (ESRC), part of UK Research and Innovation. The Millennium Cohort Study is funded by grants to the Centre for Longitudinal Studies at the Institute of Education from the Economic and Social Research Council and a consortium of government department.

The Australian arm of the BEACHES study was funded by the Australian National Health and Medical Research Council - UKRI-NHMRC Built Environment Prevention Research Scheme (GNT1192764). This research is supported (partially) by the Australian Government through the Australian Research Council's Centre of Excellence for Children and Families over the Life Course (Project ID CE200100025). HC is supported by an Australian National Heart Foundation Future Leader Fellowship (102549). The ORIGINS Project has received core funding support from the Telethon Perth Children's Hospital Research Fund, Joondalup Health Campus, the Paul Ramsay Foundation and the Commonwealth Government of Australia through the Channel 7 Telethon Trust. Substantial in-kind support has been provided by Telethon Kids Institute and Joondalup Health Campus.

For peer review only

References

1 Bentham J, Di Cesare M, Bilano V, et al. Worldwide trends in body-mass index, underweight, overweight, and obesity from 1975 to 2016: a pooled analysis of 2416 population-based measurement studies in 128·9 million children, adolescents, and adults. *Lancet*. 2017;390(10113).

2 WHO. Report of the Commission on Ending Childhood Obesity: implementation plan Report by the Secretariat [Internet]. 2017. Available from: http://apps.who.int/iris/bitstream/10665/204176/1/9789241510066_eng.pdf?ua=1

3 Coppinger T, Milton K, Murtagh E, et al. Global Matrix 3.0 physical activity report card for children and youth: a comparison across Europe. *Public Health*. 2020;187:150–6.

4 Christian HE, Bull FC, Middleton NJ, et al. How important is the land use mix measure in understanding walking behaviour? Results from the RESIDE study. *Int J Behav Nutr Phys Act*.

For peer review only - http://bmjopen.bmj.com/site/about/guidelines.xhtml

2011;8(1):55. Available from: http://ijbnpa.biomedcentral.com/articles/10.1186/1479-5868-8-55

5 Foster S, Wood L, Christian H, et al. Planning safer suburbs: Do changes in the built environment influence residents' perceptions of crime risk? *Soc Sci Med*. 2013;97:87–94.

6 Christian H, Knuiman M, Divitini M, et al. A Longitudinal Analysis of the Influence of the Neighborhood Environment on Recreational Walking within the Neighborhood: Results from RESIDE. *Environ Health Perspect*. 2017;125(7):077009. Available from: https://ehp.niehs.nih.gov/doi/10.1289/EHP823

7 Müller-Riemenschneider F, Pereira G, Villanueva K, et al. Neighborhood walkability and cardiometabolic risk factors in australian adults: An observational study. *BMC Public Health*. 2013;13(1):755. Available from: http://bmcpublichealth.biomedcentral.com/articles/10.1186/1471-2458-13-755

8 Bird EL, Ige JO, Pilkington P, et al. Built and natural environment planning principles for promoting health: An umbrella review. *BMC Public Health*. 2018;18(1).

9 Ortegon-Sanchez A, McEachan RRC, Albert A, et al. Measuring the Built Environment in Studies of Child Health-A Meta-Narrative Review of Associations. *Int J Environ Res Public Health*. 2021;18(20). Available from: https://pubmed.ncbi.nlm.nih.gov/34682484/

10 Nobles J, Summerbell C, Brown T, et al. A secondary analysis of the childhood obesity prevention Cochrane Review through a wider determinants of health lens: implications for research funders, researchers, policymakers and practitioners. *Int J Behav Nutr Phys Act*. 2021;18(1). Available from: https://pubmed.ncbi.nlm.nih.gov/33563281/

11 Dixon BN, Ugwoaba UA, Brockmann AN, et al. Associations between the built environment and dietary intake, physical activity, and obesity: A scoping review of reviews. *Obes Rev*. 2021;22(4):e13171. Available from: https://onlinelibrary.wiley.com/doi/10.1111/obr.13171

12 Jia P, Zou Y, Wu Z, et al. Street connectivity, physical activity, and childhood obesity: A systematic review and meta-analysis. *Obes Rev*. 2021;22(S1):e12943. Available from: https://onlinelibrary.wiley.com/doi/10.1111/obr.12943

13 Volger S, Rigassio Radler D, Rothpletz-Puglia P. Early childhood obesity prevention efforts through a life course health development perspective: A scoping review. Ells L, editor. *PLoS One*. 2018;13(12):e0209787. Available from: https://dx.plos.org/10.1371/journal.pone.0209787

For peer review only

For peer review only - http://bmjopen.bmj.com/site/about/guidelines.xhtml

14 Wu S, Ding Y, Wu F, et al. Socio-economic position as an intervention against overweight and obesity in children: A systematic review and meta-analysis. *Sci Rep*. 2015;5. Available from: https://pubmed.ncbi.nlm.nih.gov/26112253/

15 Lobstein T, Jackson-Leach R, Moodie ML, et al. Child and adolescent obesity: Part of a bigger picture [Internet]. Vol. 385, The Lancet. Lancet Publishing Group; 2015. p. 2510–20. Available from: /pmc/articles/PMC4594797/

16 Blüher M. Obesity: global epidemiology and pathogenesis [Internet]. Vol. 15, Nature Reviews Endocrinology. Nature Publishing Group; 2019. p. 288–98. Available from: https://pubmed.ncbi.nlm.nih.gov/30814686/

17 Coghlan AT, Preskill H, Tzavaras Catsambas T. An overview of appreciative inquiry in evaluation. *New Dir Eval*. 2003;2003(100):5–22. Available from: https://onlinelibrary.wiley.com/doi/full/10.1002/ev.96

18 Cooperrider DL, Whitney DK. Appreciative inquiry : a positive revolution in change [Internet]. . ReadHowYouWant. com; 111 p. Available from: https://books.google.com/books/about/Appreciative_Inquiry.html?id=bJBgktz2Oa8C

19 Public Health Wales. Creating healthier places and spaces for our present and future generations [Internet]. 2018. Available from: www.nationalarchives.gov.uk/doc/open-government-licence/version/3/

20 Klepac Pogrmilovic B, O'Sullivan G, Milton K, et al. The development of the Comprehensive Analysis of Policy on Physical Activity (CAPPA) framework. *Int J Behav Nutr Phys Act*. 2019;16(1):1–15. Available from: https://ijbnpa.biomedcentral.com/articles/10.1186/s12966-019-0822-5

21 Mizen A, Song J, Fry R, et al. Longitudinal access and exposure to green-blue spaces and individual-level mental health and well-being: Protocol for a longitudinal, population-wide record-linked natural experiment. *BMJ Open*. 2019;9(4). Available from: https://pubmed.ncbi.nlm.nih.gov/31005938/

22 Mavoa S, Bagheri N, Koohsari MJ, et al. How Do Neighbourhood Definitions Influence the Associations between Built Environment and Physical Activity? *Int J Environ Res Public Health*. 2019;16(9):1501. Available from: https://www.mdpi.com/1660-4601/16/9/1501/htm

23 Noardo F, Lingua A, Aicardi I, et al. Cartographic data harmonisation for a cross-border project development. *Appl Geomatics*. 2016;8(3):133–50.

For peer review only

For peer review only - http://bmjopen.bmj.com/site/about/guidelines.xhtml

24 Fry R, Orford S, Rodgers S, et al. A best practice framework to measure spatial variation in alcohol availability. *Environ Plan B Urban Anal City Sci*. 2020;47(3):381–99. Available from: http://journals.sagepub.com/doi/10.1177/2399808318773761

25 Hyatt M, Rodgers SE, Paranjothy S, et al. The wales electronic cohort for children (WECC) study. *Arch Dis Child - Fetal Neonatal Ed*. 2011;96(Suppl 1):Fa18–Fa18. Available from: https://fn.bmj.com/content/96/Suppl_1/Fa18.1

26 Connelly R, Platt L. Cohort profile: UK Millennium Cohort Study (MCS). *Int J Epidemiol*. 2014;43(6):1719–25.

27 Fitzsimons E BD. Obesity prevalence and its inequality from childhood to adolescence [Internet]. Available from: https://cls.ucl.ac.uk/wp-content/uploads/2020/12/CLS-briefing-paper-Obesity-prevalence-MCS-age-17-initial-findings.pdf

28 Griffiths LJ, Cortina-Borja M, Sera F, et al. How active are our children? Findings from the Millennium Cohort Study. *BMJ Open*. 2013;3(8):e002893. Available from: https://bmjopen.bmj.com/content/3/8/e002893

29 Fluharty M, Villadsen A, Kandola A, et al. Physical activity across age and study: a guide to data in six CLOSER studies Resource report. 2020.

30 Maddock J, O’neill D, Robinson S, et al. A guide to the dietary data in eight CLOSER studies Resource report. 2020.

31 Christian H, Rosenberg M, Trost S, et al. A snapshot of the PLAYCE project: Findings from the Western Australian PLAY Spaces and Environments for Children’s Physical Activity Study. Supportive Childcare Environments for Physical Activity in the Early Years. Perth, Western Australia: The Universit. 2018 Dec.

32 Christian H, Maitland C, Enkel S, et al. Influence of the day care, home and neighbourhood environment on young children’s physical activity and health: protocol for the PLAYCE observational study. *BMJ Open*. 2016;6(12):14058. Available from: http://dx.doi.org/10.1136/bmjopen-2016-014058

33 Pate RR, O’Neill JR, Mitchell J. Measurement of physical activity in preschool children. *Med Sci Sports Exerc*. 2010;42(3):508–12.

34 Hinkley T, Salmon J, Okely AD, et al. The HAPPY study: development and reliability of a parent survey to assess correlates of preschool children’s physical activity. *J Sci Med Sport*. 2012;15(5):407–17.

For peer review only

For peer review only - http://bmjopen.bmj.com/site/about/guidelines.xhtml

35 Burdette HL, Whitaker RC, Daniels SR. Parental report of outdoor playtime as a measure of physical activity in preschool-aged children. *Arch Pediatr Adolesc Med*. 2004;158(4):353–7.

36 Hendrie GA, Viner Smith E, Golley RK. The reliability and relative validity of a diet index score for 4-11-year-old children derived from a parent-reported short food survey. *Public Health Nutr*. 2014;17(7):1486–97. Available from: https://pubmed.ncbi.nlm.nih.gov/23830074/

37 Silva DT, Hagemann E, Davis JA, et al. Introducing the ORIGINS project: a community-based interventional birth cohort. *Rev Environ Health*. 2020;35(3):281–93. Available from: https://www.degruyter.com/document/doi/10.1515/reveh-2020-0057/html

38 Collins CE, Burrows TL, Truby H, et al. Comparison of energy intake in toddlers assessed by food frequency questionnaire and total energy expenditure measured by the doubly labeled water method. *J Acad Nutr Diet*. 2013;113(3):459–63. Available from: https://pubmed.ncbi.nlm.nih.gov/23317500/

39 Soloff C, Lawrence D, Misson S, et al. The Longitudinal Study of Australian Children: an Australian Government initiative - Wave 1 weighting and non-response. 2006.

40 Yu M, Baxter J. 5. Australian children's screen time and participation in extracurricular activities - LSAC Annual Statistical Report 2015.

41 Lyons RA, Jones KH, John G, et al. The SAIL databank: Linking multiple health and social care datasets. *BMC Med Inform Decis Mak*. 2009;9(1):3. Available from: https://bmcmedinformdecismak.biomedcentral.com/articles/10.1186/1472-6947-9-3

42 Ford D V., Jones KH, Verplancke JP, et al. The SAIL Databank: Building a national architecture for e-health research and evaluation. *BMC Health Serv Res*. 2009;9(1):157.

43 Johnson RD, Griffiths LJ, Hollinghurst JP, et al. Deriving household composition using population-scale electronic health record data—A reproducible methodology. *PLoS One*. 2021;16(3):e0248195. Available from: https://journals.plos.org/plosone/article?id=10.1371/journal.pone.0248195

44 Hooper P, Foster S, Bull F, et al. Living liveable? RESIDE's evaluation of the "Liveable Neighborhoods" planning policy on the health supportive behaviors and wellbeing of residents in Perth, Western Australia. *SSM - Popul Heal*. 2020;10:100538.

45 Davies HT, Powell AE, Nutley SM. Mobilising knowledge to improve UK health care: learning from other countries and other sectors – a multimethod mapping study. *Heal Serv Deliv Res*. 2015;3(27):1–190. Available from: https://www.ncbi.nlm.nih.gov/books/NBK299400/

For peer review only

For peer review only - http://bmjopen.bmj.com/site/about/guidelines.xhtml

Figure Legend

Figure 1 - BEACHES project workflow.

Additional Information

Acknowledgements

This study makes use of anonymised data held in the Secure Anonymised Information Linkage (SAIL) Databank. We would like to acknowledge all the data providers who make anonymised data available for research. We also thank all the Millennium Cohort families for their participation, the director of the Millennium Cohort Study and colleagues in the management team at the Centre for Longitudinal Studies, UCL Institute of Education. Partners for the Australian arm include the Western Australian (WA) Department of Local Government, Sport and Cultural Industries, WA Department of Health, WA Department of Transport, WA Local Government Association, Australian Childcare Alliance, Nature Play Australia, Heart Foundation, Cancer Council WA, Goodstart Early Learning, and Hames Sharley. The ORIGINS Project is only possible because of the commitment of the families in ORIGINS. We are grateful to all the participants, health professionals and researchers who support the project. We would also like to acknowledge and thank the following teams and individuals who have made The ORIGINS Project possible: The ORIGINS Project team; CEO Dr Kempton Cowan, executive staff and obstetric, neonatal and paediatric teams, Joondalup Health Campus (JHC); Director Professor Jonathan Carapetis and executive staff, Telethon Kids Institute; Mayor Tracey Roberts, City of Wanneroo; Mayor Albert Jacobs, City of Joondalup; Professor Fiona Stanley, patron of ORIGINS; members of ORIGINS Community Reference and Participant Reference Groups; Research Interest Groups and the ORIGINS Scientific Committee. BEACHES is a sub-project of The ORIGINS Project. This unique long-term study, a collaboration between Telethon Kids Institute and Joondalup Health Campus, is one of the most comprehensive studies of pregnant women and their families in Australia to date, recruiting 10,000 families over a decade from the Joondalup and Wanneroo communities of Western Australia.

For peer review only - http://bmjopen.bmj.com/site/about/guidelines.xhtml

640

641 **Disclaimer**

642 The views expressed in this paper are those of the authors and not necessarily those of the funding

643 bodies, the NHS, The NIHR, or the Department of Health and Social Care.

644

645 **Acronyms**

BMI	Body Mass Index
CMPW	Child Measurement Programme for Wales
EVI	Enhanced Vegetation Index
GIS	Geographic Information Systems
GNAF	Geoscape Geocoded National Address File
LSAC	Longitudinal Study of Australian Children
MCS	Millennium Cohort Study
NCD	Non-Communicable Disease
NDVI	Normalised Difference Vegetation Index
ONS	Office for National Statistics
OS	Ordnance Survey
OSM	Open Street Map
PLAYCE	PLAY Spaces & Environments for Children's Physical Activity
RALF	Residential Anonymised Linking Field
SAIL	Secure Anonymised Information Linkage
SES	Socio-economic status
WA	Western Australia
WECC	Wales Electronic Cohort for Children
UK	United Kingdom
UPRN	Unique Property Reference Number

646

For peer review only - http://bmjopen.bmj.com/site/about/guidelines.xhtml

For peer review only

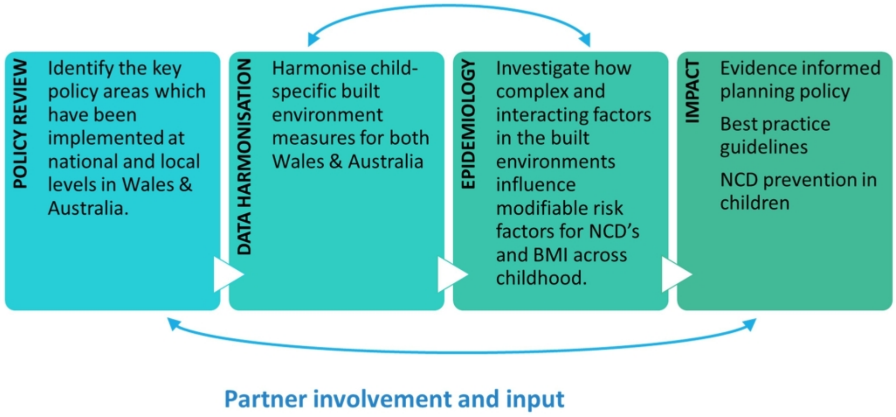

BEACHES project workflow

297x209mm (300 x 300 DPI)

bmjopen-2022-061978 - "Built Environment and Child Health in Wales and Australia (BEACHES): a study protocol"

STROBE Statement—checklist of items that should be included in reports of observational studies

	Item No.	Recommendation	Page No.	Relevant text from manuscript
**Title and abstract**	1	(*a*) Indicate the study's design with a commonly used term in the title or the abstract	1<br>3	Title - Line: 1<br>Abstract - Lines: 67
		(*b*) Provide in the abstract an informative and balanced summary of what was done and what was found	3	Lines: 67-74<br>As this is a protocol, we also report how findings will be disseminated in lines 79-83
**Introduction**				
Background/rationale	2	Explain the scientific background and rationale for the investigation being reported	4-5	Lines 101-136
Objectives	3	State specific objectives, including any prespecified hypotheses	5-6	Lines: 139-152
**Methods**				
Study design	4	Present key elements of study design early in the paper	6	Lines: 156-166
Setting	5	Describe the setting, locations, and relevant dates, including periods of recruitment, exposure, follow-up, and data collection	6/10	Lines: 156-166; Table 2
Participants	6	(*a*) *Cohort study*—Give the eligibility criteria, and the sources and methods of selection of participants. Describe methods of follow-up<br>*Case-control study*—Give the eligibility criteria, and the sources and methods of case ascertainment and control selection. Give the rationale for the choice of cases and controls<br>*Cross-sectional study*—Give the eligibility criteria, and the sources and methods of selection of participants	10-12	Description of cohort data<br>Lines 245-326
		(*b*) *Cohort study*—For matched studies, give matching criteria and number of exposed and unexposed<br>*Case-control study*—For matched studies, give matching criteria and the number of controls per case	n/a	
Variables	7	Clearly define all outcomes, exposures, predictors, potential confounders, and effect modifiers. Give diagnostic criteria, if applicable	13	Covariates - Lines: 352-354

For peer review only - http://bmjopen.bmj.com/site/about/guidelines.xhtml

Data sources/ measurement	8*	For each variable of interest, give sources of data and details of methods of assessment (measurement). Describe comparability of assessment methods if there is more than one group	7-9<br>10-12	Built Environment - Lines:200-239<br>Health data - Lines: 245-326
Bias	9	Describe any efforts to address potential sources of bias	n/a	
Study size	10	Explain how the study size was arrived at	10	Table 2 details cohort sizes
Quantitative variables	11	Explain how quantitative variables were handled in the analyses. If applicable, describe which groupings were chosen and why	n/a	
Statistical methods	12	(*a*) Describe all statistical methods, including those used to control for confounding	13<br>13-14	Data linkage - Lines:328-346<br>Statistical analyses - Lines: 348-382
		(*b*) Describe any methods used to examine subgroups and interactions		
		(*c*) Explain how missing data were addressed	n/a	
		(*d*) *Cohort study*—If applicable, explain how loss to follow-up was addressed<br>*Case-control study*—If applicable, explain how matching of cases and controls was addressed<br>*Cross-sectional study*—If applicable, describe analytical methods taking account of sampling strategy	n/a	
		(*e*) Describe any sensitivity analyses	13	Analysis of different population groups - Lines: 354-355
**Results**				
Participants	13*	(a) Report numbers of individuals at each stage of study—eg numbers potentially eligible, examined for eligibility, confirmed eligible, included in the study, completing follow-up, and analysed	n/a	
		(b) Give reasons for non-participation at each stage	n/a	
		(c) Consider use of a flow diagram	n/a	
Descriptive data	14*	(a) Give characteristics of study participants (eg demographic, clinical, social) and information on exposures and potential confounders	n/a	
		(b) Indicate number of participants with missing data for each variable of interest	n/a	
		(c) *Cohort study*—Summarise follow-up time (eg, average and total amount)	n/a	
Outcome data	15*	*Cohort study*—Report numbers of outcome events or summary measures over time	n/a	

For peer review only - http://bmjopen.bmj.com/site/about/guidelines.xhtml

		Case-control study—Report numbers in each exposure category, or summary measures of exposure	n/a	
		Cross-sectional study—Report numbers of outcome events or summary measures		
Main results	16	(*a*) Give unadjusted estimates and, if applicable, confounder-adjusted estimates and their precision (eg, 95% confidence interval). Make clear which confounders were adjusted for and why they were included	n/a	
		(*b*) Report category boundaries when continuous variables were categorized	n/a	
		(*c*) If relevant, consider translating estimates of relative risk into absolute risk for a meaningful time period	n/a	
Other analyses	17	Report other analyses done—eg analyses of subgroups and interactions, and sensitivity analyses	n/a	
**Discussion**				
Key results	18	Summarise key results with reference to study objectives	n/a	
Limitations	19	Discuss limitations of the study, taking into account sources of potential bias or imprecision. Discuss both direction and magnitude of any potential bias	4	Strengths and limitations of the study – Lines: 87-98
Interpretation	20	Give a cautious overall interpretation of results considering objectives, limitations, multiplicity of analyses, results from similar studies, and other relevant evidence	n/a	
Generalisability	21	Discuss the generalisability (external validity) of the study results	15-16	Discussion - Lines: 421-437
**Other information**				
Funding	22	Give the source of funding and the role of the funders for the present study and, if applicable, for the original study on which the present article is based	16	Funding - Lines: 453-469

*Give information separately for cases and controls in case-control studies and, if applicable, for exposed and unexposed groups in cohort and cross-sectional studies.

Note: An Explanation and Elaboration article discusses each checklist item and gives methodological background and published examples of transparent reporting. The STROBE checklist is best used in conjunction with this article (freely available on the Web sites of PLoS Medicine at http://www.plosmedicine.org/, Annals of Internal Medicine at http://www.annals.org/, and Epidemiology at http://www.epidem.com/). Information on the STROBE Initiative is available at www.strobe-statement.org.

For peer review only

BMJ Open

Built Environment and Child Health in Wales and Australia (BEACHES): a study protocol

Journal:	*BMJ Open*
Manuscript ID	bmjopen-2022-061978.R2
Article Type:	Protocol
Date Submitted by the Author:	01-Sep-2022
Complete List of Authors:	Pedrick-Case, Rebecca; Swansea University Medical School, Population Data Science<br>Bailey, Rowena; Swansea University Medical School, Population Data Science<br>Beck, Ben; Monash University, School of Public Health and Preventive Medicine<br>Beesley, Bridget; The University of Western Australia, UWA School of Agriculture and Environment<br>Boruff, Bryan; The University of Western Australia, UWA School of Agriculture and Environment<br>Brophy, Sinead; Swansea University Medical School, Population Data Science<br>Cross, Donna; The University of Western Australia, Telethon Kids Institute; The University of Western Australia, School of Population and Global Health<br>Dhamrait, Gursimran; The University of Western Australia, Telethon Kids Institute; The University of Western Australia, School of Population and Global Health<br>Duncan, John; University of Western Australia, UWA School of Agriculture and Environment<br>Gething, Peter; The University of Western Australia, Telethon Kids Institute; Curtin University, Faculty of Health Sciences<br>Johnson, Rhodri; Swansea University Medical School, Population Data Science<br>Lyons, Ronan; Swansea University Medical School, Population Data Science<br>Mizen, Amy; Swansea University Medical School, Population Data Science<br>Murray, Kevin; University of Western Australia, School of Population and Global Health<br>Pouliou, Theodora; Swansea University Medical School, Population Data Science<br>Rafferty, James; Swansea University Medical School, Population Data Science<br>Robinson, Trina; The University of Western Australia, Telethon Kids Institute<br>Rosenberg, Michael; University of Western Australia, School of Human Sciences<br>Schipperijn, Jasper; University of Souther Denmark, Department of Sports Science and Clinical Biomechanics<br>Thompson, Daniel; Swansea University Medical School, Population Data

	Science<br>Trost, Stewart; The University of Queensland, School of Human Movement and Nutrition Sciences<br>Watkins, Alan; Swansea University Medical School<br>Stratton, Gareth; Swansea University, Research Centre in Applied Sports, Technology, Exercise and Medicine<br>Fry, Richard; Swansea University Medical School, Population Data Science<br>Christian, Hayley; The University of Western Australia, Telethon Kids Institute; The University of Western Australia, School of Population and Global Health<br>Griffiths, Lucy ; Swansea University, Swansea University Medical School

**Primary Subject Heading**:	Epidemiology
Secondary Subject Heading:	Epidemiology
Keywords:	EPIDEMIOLOGY, PAEDIATRICS, PREVENTIVE MEDICINE, PUBLIC HEALTH

SCHOLARONE™
Manuscripts

Built Environment and Child Health in Wales and Australia (BEACHES): a study protocol

Rebecca Pedrick-Case[1], Rowena Bailey[1], Ben Beck[2], Bridget Beesley[3], Bryan Boruff[3], Sinead Brophy[1], Donna Cross [4,5], Gursimran Dhamrait [4,5], John Duncan[3], Peter Gething[4,6], Rhodri D Johnson[1], Ronan A Lyons[1], Amy Mizen[1], Kevin Murray[5], Theodora Pouliou[1], James Rafferty[1], Trina Robinson[4],Michael Rosenberg[7], Jasper Schipperijn[8], Daniel A Thompson[1], Stewart Trost[9], Alan Watkins[1], Gareth Stratton[10,#], Richard Fry[1,#], Hayley Christian [4,5,#], Lucy J Griffiths[1,#,*]

[1] Population Data Science, Swansea University Medical School, Swansea, UK.

[2] School of Public Health and Preventive Medicine, Monash University, Melbourne, Australia.

[3] UWA School of Agriculture and Environment, University of Western Australia, Perth, Australia.

[4] Telethon Kids Institute, University of Western Australia, Perth, Australia.

[5] School of Population and Global Health, University of Western Australia, Perth, Australia.

[6] Faculty of Health Sciences, Curtin University, Perth, Australia.

[7] School of Human Sciences, University of Western Australia, Perth, Australia.

[8] Department of Sports Science and Clinical Biomechanics, University of Southern Denmark, Odense, Denmark.

[9] School of Human Movement and Nutrition Sciences, University of Queensland, Brisbane, Australia.

[10] Research Centre in Applied Sports, Technology, Exercise and Medicine (A-STEM), Swansea University, Swansea, UK.

* Corresponding author: Dr Lucy Griffiths, Swansea University Medical School, Swansea, University, Swansea, UK; lucy.griffiths@swansea.ac.uk

# Joint senior authors

ORCID IDs

For peer review only

For peer review only - http://bmjopen.bmj.com/site/about/guidelines.xhtml

Rebecca Pedrick-Case: 0000-0003-2307-1808

Rowena Bailey: 0000-0003-2409-2045

Ben Beck: 0000-0003-3262-5956

Bridget Beesley: 0000-0003-0432-3766

Bryan Boruff: 0000-0001-6693-0671

Sinead Brophy: 0000-0001-7417-2858

Donna Cross: 0000-0001-6217-5058

Gursimran K Dhamrait: 0000-0002-5191-211X

John Duncan: 0000-0001-9752-1002

Peter Gething: 0000-0001-6759-5449

Rhodri D Johnson: 0000-0001-9636-0753

Ronan A Lyons: 0000-0001-5225-000X

Amy Mizen: 0000-0001-7516-6767

Kevin Murray: 0000-0002-8856-6046

Theodora Pouliou: 0000-0002-1162-1174

James Rafferty: 0000-0002-1667-7265

Trina Robinson: 0000-0003-2638-958X

Michael Rosenberg: 0000-0002-9950-5775

Jasper Schipperijn: 0000-0002-6558-7610

Daniel A Thompson: 0000-0002-1769-2870

Stewart Trost: 0000-0001-9587-3944

Alan Watkins: 0000-0003-3804-1943

Rich Fry: 0000-0002-7968-6679

Gareth Stratton: 0000-0001-5618-0803

Hayley Christian: 0000-0001-8486-5746

Lucy J Griffiths: 0000-0001-9230-624X

Word Count: 3568/4,000 words

Keywords: Non-communicable disease, childhood obesity, built environment, linked data, cohort studies, ORIGINS Project, PLAYCE Cohort Study, Longitudinal Study of Australian Children, Millennium Cohort Study, SAIL Databank.

For peer review only

For peer review only - http://bmjopen.bmj.com/site/about/guidelines.xhtml

Abstract

Introduction: Childhood obesity and physical inactivity are two of the most significant modifiable risk factors for the prevention of non-communicable diseases (NCDs). Yet, a third of children in Wales and Australia are overweight or obese, and only 20% of UK and Australian children are sufficiently active. The purpose of this study is to identify and understand how complex and interacting factors in the built environment influence modifiable risk factors for NCDs across childhood.

Methods & Analysis: This is an observational study using data from five established cohorts from Wales and Australia: 1) Wales Electronic Cohort for Children (WECC); 2) Millennium Cohort Study (MCS); 3) PLAY Spaces & Environments for Children's Physical Activity (PLAYCE) study; 4) the ORIGINS Project; and 5) The Growing Up in Australia: the Longitudinal Study of Australian Children (LSAC). The study will incorporate a comprehensive suite of longitudinal quantitative data (surveys, anthropometry, accelerometery, and Geographic Information Systems (GIS) data) to understand how the built environment influences children's modifiable risk factors for NCDs (Body Mass Index (BMI), physical activity, sedentary behaviour, and diet).

Ethics & Dissemination: This study has received the following approvals: The University of Western Australia Human Research Ethics Committee (2020/ET000353), Ramsay Human Research Ethics Committee (under review), and Swansea University Information Governance Review Panel approval (Project ID: 1001). Findings will be reported to the following: 1) funding bodies, research institutes and hospitals supporting the BEACHES project; 2) parents and children; 3) school management teams; 4) existing and new industry partner networks; 5) federal, state, and local governments to inform policy; as well as 6) presented at local, national, and international conferences; and 7) disseminated by peer-review publications.

For peer review only - http://bmjopen.bmj.com/site/about/guidelines.xhtml

For peer review only

Article Summary

Strengths and limitations of this study

- The BEACHES project uses large representative samples of children from five Wales and Australian cohort studies.
- Standardised built environment measures will be applied across Wales and Australia and linked to the cohort study data at the individual-level.
- The contrasting timepoints, climates, geographies, and policy approaches in Wales and Australia will provide stronger evidence of the causal pathways between the built environment and child health.
- The use of existing cohort datasets can limit the use of consistent health outcomes across studies.
- Analyses using routinely recorded data (Wales) may omit some unknown confounders, thereby introducing a moderate level of bias due to confounding.

Introduction

Childhood obesity and physical inactivity are two of the most significant modifiable risk factors for non-communicable disease (NCD) prevention in children.[1] The 2017 Commission on Ending Childhood Obesity emphasised that the prevention of modifiable risk factors for NCDs should start as early as possible.[2] Yet only 20% of UK and Australian children are sufficiently active, and over 60% engage in excessive sedentary time, with a third overweight or obese.[3]

The built environment in which we live is integral to human health. Research has shown that residing in 'liveable' neighbourhoods characterised by: good access to shops, services, quality parks, connected streets to facilitate walking, sufficient residential densities to support public transport services and local businesses, minimal crime and traffic, and social connectedness opportunities are associated with improved health outcomes.[4–9]. Despite the increasing evidence of the association between the built environment and physical activity, there is still a paucity of longitudinal research examining the role of the built environment in promoting child health.[10,11] Critical evidence gaps for young people include: 1) causal evidence of the overall impact the built environment has on children's modifiable risk factors for NCDs (i.e., physical inactivity, sedentariness, and poor diet);[11,12] 2) the specific effect

For peer review only - http://bmjopen.bmj.com/site/about/guidelines.xhtml

of individual, familial, and combinations of built environment attributes; 3) how these effects vary across different ages of children;[13] 4) how these effects are moderated by socioeconomic status;[14] 5) the mediating role of physical activity and healthy eating on the relationship between the built environment and child obesity;[15] and 6) how the influence of the built environment on children's modifiable risk factors for NCDs varies by different geographical locations.[16] These evidence gaps hinder the formulation of specific, actionable policies to improve the built environment for child health.

The Built Environment and Child Health in Wales and Australia (BEACHES) project is a collaboration between academic institutions in Wales and Australia to identify and understand how complex and interacting factors in the built environment influence modifiable risk factors for NCDs across childhood. This project aims to address identified evidence gaps by using Wales and Australian longitudinal population linked and cohort data to identify and understand how complex and interacting factors in the built environment influence modifiable risk factors for NCDs (physical inactivity, sedentariness, poor diet) across childhood. Five large Welsh and Australian cohort studies, with detailed anthropometric, physical (in)-activity, diet, and contextual data for more than one million children will be used. We will use the highest quality available spatial data and geospatial techniques to construct an internationally standardised set of metrics, such as walkability, that characterise the built environments each child has inhabited during childhood. A statistical modelling framework will be used to quantify the influence that different built environment characteristics have on children's BMI, and the respective roles of physical activity, sedentary behaviour, and diet in this relationship.

Objectives

The purpose of this study is to identify and understand how complex and interacting factors in the built environment influence modifiable risk factors for NCDs across childhood. The objectives are to:

1. Develop a comprehensive Geographic Information Systems (GIS) model of child-specific built environment characteristics using standardised methods for Wales and Australia,
2. Link standardised GIS models of the built environment to e-cohort and standard cohort data for children in Wales and Australia,
3. Determine the direct and indirect (and mediating) relationships between the built environment and: a) children's modifiable risk factors for NCDs (physical activity, sedentary time, and diet), and b) obesity,

For peer review only - http://bmjopen.bmj.com/site/about/guidelines.xhtml

For peer review only

4. Identify how relationships between the built environment and these NCD risk factors vary by children's socio-economic position and geographic location (across and within Wales and Australia), and
5. Produce evidence which policymakers and other stakeholders can use to modify the built environment to enable physical activity and reduce childhood obesity.

Methods and analysis

This multi-site study (funding period: 2020-2023) will use population-level cohort data (including but not limited to surveys, anthropometry, accelerometry, GIS data, and biological samples) to address these five objectives (Figure 1). We will review the policy landscape and literature on the key built environment and child health-related policies at national and local levels in Wales and Australia. Based on the findings, we will curate and harmonise environmental data that will be used to develop high resolution spatial models of the built environment. These built environment measures will be linked with cohort and population-level health data (Wales) to identify and understand how complex and interacting factors in the built environment influence modifiable risk factors for NCDs and BMI across childhood using a series of covariate-adjusted, multi-level regression models. Finally, we will communicate to policymakers and planners which modifiable aspects of the built environment may contribute to a reduction in risk factors for NCDs and BMI across childhood.

INSERT Figure 1

Policy & Literature Review

We will actively engage with stakeholders and carry out a policy landscape review (for the duration of the cohort studies) to examine key built environment and child health-related policies at national and local levels in Wales and Australia. Findings will provide the overall context for policy gaps and landscapes, and the co-design of research to address these gaps, develop best practice guidelines and overall, reduce NCDs in children.

Our stakeholders include health service commissioners, government departments of planning, transport and health, planning officials, urban planners, building industry, developers and key advocacy non-governmental organisations, as well as third sector and voluntary agencies.

For peer review only

In Wales, we will engage with our stakeholders at three time points throughout the project. These include, for example, Public Health Wales, Active Healthy Kids Wales and Play Wales. We will use appropriate frameworks (e.g. 'appreciative inquiry') [17,18] to inform and interpret our quantitative research findings into stakeholder engagement activities. We will map out policies and key legislation across Wales using the six priority areas of the built and natural environment that Public Health Wales has published in the 'Creating healthier places and spaces for our present and future generations 'report.[19]

In Australia, we plan to meet our government and non-government partners quarterly each year for the length of the grant. These include the Western Australian (WA) Department of Local Government, Sport and Cultural Industries, WA Department of Health, WA Department of Transport, WA Local Government Association, Australian Childcare Alliance, Nature Play Australia, Heart Foundation, The PLAY Spaces and Environments for Children's Physical Activity (PLAYCE) partners, Cancer Council WA, Goodstart Early Learning, and Hames Sharley. With our partners, we will conduct a policy analysis to investigate how Western Australian and national policies address the health of children through the built environment's influence on obesity and the modifiable risk factors for obesity, physical activity, sedentary behaviour, and diet. Policy analysis is crucial to achieving reforms in health promotion by raising awareness of current policy gaps and opportunities and demonstrating successful policy related actions being taken across the system. The Comprehensive Analysis of Policy on Physical Activity (CAPPA) framework will be used to guide the analysis.[20]

Built Environment Measures

GIS-derived built environment measures will be calculated at the residential address level for Wales and Australia. Table 1 outlines the standardised built environment measures that will be created for all Wales and Australian datasets. We will use rich vector spatial datasets which define land utilisation (e.g. building footprint and height, cadastre, road centreline and reserve, and points of interest), planning data (e.g. active travel routes, open space assessments, fast food outlets) and Earth Observation data (e.g. Landsat, Sentinel, Aerial Photography). We have developed a broad range of longitudinal data and methods which characterise the built environment of the two study areas (Table 2). These include neighbourhood walkability, garden size/home outdoor area, blue-green space availability,[21] access to services and facilities, and fast-food outlets around the home and school.

Annual acquisition and cataloguing of Western Australian GIS data has occurred since 2005, first under the guise of the former University of Western Australia Centre for the Built Environment and Health and currently as part of the PLAY Spaces & Environments for Children's Physical Activity

For peer review only - http://bmjopen.bmj.com/site/about/guidelines.xhtml

(PLAYCE) cohort study. Consistent historic GIS data made available to the BEACHES project has been obtained from Landgate, Western Australia's State geospatial data provider (e.g., road centreline, cadastre, street address and imagery), as well as high resolution four-band aerial imagery from the Urban Monitor [22] aerial imagery acquisition program (2007, 2009, 2014, 2016, 2018 and 2020), Australian Bureau of Statistics bi-decadal census available on-line since 1996 and historic satellite imagery (Table 1).

In Wales, these data will be sourced, where possible, from open data (e.g., OpenStreetMap) but will also be supplemented with existing Ordnance Survey data including MasterMap (temporal coverage: 1999-present day), MasterMap Highways (1997-present day), MasterMap Imagery Layer (1998-present day) and AddressBase Premium (1970-present day), which are made available to the project via the Public Sector GeoSpatial Agreement and Higher Education Agreements with Ordnance Survey. These data, coupled with opensource Landsat and OpenStreetMap data will allow us to build a rich temporal coverage of the built environment to align with the linked health data.

In previous studies of built-environment measures for physical activity, how the neighbourhood is defined has greatly influenced the results.[23] For example, access to green and blue spaces or destinations (retail and food outlets) can be based on distance from a child's residence measured as a straight-line (Euclidean distance) from a household to a destination or based on movement along a road network, with often very different results. Therefore, the GIS and epidemiological teams will work together closely with a 'behaviour-led approach' to define the neighbourhood sizes most likely to correlate with the behaviour of interest by age group. For example, active travel from home to school.

Harmonisation of Built Environment Measures

As far as reasonably possible, the data, spatial analytic approaches, and built environment measures will be standardised and harmonised across the Welsh and Australian study cohorts to enable the calculation of comparable measures. This will involve using temporally aligned GIS and cohort data (i.e., built-environment metrics generated using spatially referenced data relating to the epidemiological timepoint of interest), age-specific built environment profiles and neighbourhood characterisations. In addition, feature level metadata inherent to baseline GIS datasets allows for extraction of historic representations of geographic features to match cohort data collection timepoints. Where temporal mismatches occur, we will explore methods to impute exposure metrics using the data available, to align with the appropriate time points. Standardisation and harmonisation will increase confidence in the comparability of measures by minimising potential errors from heterogeneity in data collection procedures, software or methodological approaches; and

is particularly important in projects between two countries where definitions, symbology, and projection in source cartographic data will vary.[24]

Whilst both teams have access to a rich history of local data and built environment measure development, where feasible, we will use open data (e.g., OpenStreetMap) and open-source software, such as QGIS, SQL, Python or R, to reduce barriers to reproducing this research in other contexts, thus increasing the potential impact. Methods and code will be made available in future publications, and variations in terminology will be mapped out in a typology with definitions. This will help us to identify where broader policy approaches can be taken and where more contextually specific policies will need to be implemented.[25]

For peer review only

Table 1. Wales and Australian Standardised Measures of the Built Environment.

Built Environment Feature	Indicative BE Variables	Data Source	
		**Wales**	**Australia**
**Outdoor home environment**	Home garden/yard space & vegetation	Aerial imagery, NDVI/EVI	Aerial imagery, NDVI/EVI, Urban Monitor, Landgate (Cadastre)
**Child-relevant walkability**	Road traffic exposure, dwelling density, residence type, street connectivity, one-way node count, land use mix, 'local living score', public transport stops	OS Master Map, OSM	Landgate, Main Roads Western Australia, Western Australian Public Transport Authority, Valuer Generals Office, OSM
**Child-relevant green/blue space**	Public open space type (park, school grounds, natural, club/playgrounds, residual greenspace), park size category (Pocket/small, medium/large, district/regional), park features and attractiveness, vegetation, blue-space (distance to coast)	OS Master Map & Greenspace, OSM	University of Western Australia – Centre for the Built Environment and Health, Landgate
**Child-relevant destinations and features**	Playgroups, childcare centres, before and after school care services, kindergarten/nurseries, primary school, secondary school, other	Local authority data, OSM	Playgroup WA Inc, Western Australian Government Department of Local Government, Sport and Cultural Industries' Department of Education, Department of Transport, Department of Planning, Lands and Heritage, Department of Health
**Socioeconomic status**	Such as household level of income.	WIMD, individual-level SES data in SAIL databank	Socio-Economic Indexes for Areas (SEIFA)
**Household location**	Required to derive other measures	UPRN (OS), linked to RALF within SAIL database.	Property Street Address (PSA), GNAF

NDVI: Normalised Difference Vegetation Index; EVI: Enhanced Vegetation Index, OSM: Open Street Map, OS: Ordnance Survey, ONS: Office for National Statistics, SAIL: Secure Anonymised Information Linkage; UPRN: Unique Property Reference Number, RALF: Residential Anonymised Linking Field, GNAF: Geoscape Geocoded National Address File, SES: socio-economic status.

For peer review only - http://bmjopen.bmj.com/site/about/guidelines.xhtml

Data Sources, Linkage and Statistical Analysis (Epidemiology)

Built environment models (see above) will be linked with child cohort data to investigate how complex and interacting factors in the built environments may influence modifiable risk factors for NCD's and BMI across childhood.

Data Sources

Five large Welsh and Australian cohort studies will be used (Table 2). They include: 1) the Wales Electronic Cohort for Children (WECC); 2) the Millennium Cohort Study (MCS Wales); 3) the PLAY Spaces & Environments for Children's Physical Activity (PLAYCE) study (Australia); 4) the ORIGINS Project (Australia); and 5) The Growing Up in Australia: The Longitudinal Study of Australian Children (LSAC). Further details about each of the datasets are provided below. The age range covers both childhood and adolescence, with multiple data collection dates for each cohort study.

Table 2. Overview of Wales and Australian health datasets.

Characteristics	Datasets & Location				
	**WECC (Wales)**	**MCS (Wales)**	**PLAYCE (AU)**	**ORIGINS (AU)**	**LSAC (AU)**
**Age**	0-17	5-14	2-9	1-3	8-17
**Sample size**	1.3 million*	1,699-2,181	2,028	2,036	3,645
**No. time points**	Dynamic	4	3	2	5
**Collection years**	1990-ongoing**	2006-2015	2015-2022	2018-2022	2008-2018
**BMI**	□	□	□	□	□
**Physical Activity**	X	□	□	□	□
**Sedentary time**	X	□	□	□	□
**Diet**	X	□	□	□	□
**Confounders**	□	□	□	□	□
**Geolocation**	Home, school	Home, School	Home, childcare, school	Home	Home

AU: Australia, WECC: Wales Electronic Cohort for Children, MSC: Millennium Cohort Study, PLAYCE: PLAY Spaces and Environments for Children's Physical Activity, LSAC: The Longitudinal Study of Australian Children. * Cohort size for the analyses will be smaller (as described). ** As this is routine data it is continually collected; however, the observation period for analysis will be selected on availability of data.

Wales

<u>The Wales Electronic Cohort for Children (WECC)</u>

WECC is a total population anonymised electronic cohort study of all children born and living in Wales since 1990 (Table 2) (n=1.3 million). The WECC includes information for all live births (approximately 35,000 births per year), Welsh resident mother at birth, or a child who becomes a resident in Wales as identified by registering with a GP practice in Wales. The primary analyses for this study will be

For peer review only - http://bmjopen.bmj.com/site/about/guidelines.xhtml

carried out on a smaller Cohort selected from WECC who also have a BMI record in the Child Measurement Programme in Wales. This collected measured height and weights of school children upon entry to primary school (aged 4-5 years) between 2011 and 2019. Approximately 244,000 children were measured during this period. In addition, this Cohort will be supplemented with population-level health data (e.g., general practitioner (GP) records) for children and young people under the age of 18 with heights, weights or BMI values in their health records (up until 2019 approximately 247,000 children have at least one measure of BMI). However, WECC also includes linkage to total population education data and to a subset of child reported measures (20,000+) including diet, physical activity/physical literacy, wellbeing, child-assessed rating of local area for safety/play. A fuller description of the WECC and its contents can be found in Hyatt et al.[26]

The Millennium Cohort Study (MCS)

The MCS is a multi-disciplinary research project following the lives of around 19,000 young people born across England, Scotland, Wales and Northern Ireland in 2000-02.[27] The original cohort comprised 18 818 children (72% of those approached) whose parents were first interviewed at home when their child was around aged 9 months. Since then, data have been collected regularly throughout childhood and adolescence. This research aims to use information relating to approximately 2,000 children and young people resident in Wales at ages 5 (2006), 7 (2008), 11 (2012) and 14 years (2015). Children's height, weight and waist circumference were measured using standard protocols. [28] Physical activity was measured using accelerometery at age 7 [29] and, across all age groups, parent-reported measures of sedentary (e.g. parent-report screen time) and physical activity behaviours (e.g. sports club and playing outside),[30,31] as well as dietary factors (e.g. snacking, breakfast consumption) and a range of covariate information relating to socio-economic and demographic factors of the cohort and both resident parents were recorded.

Australia

PLAY Spaces & Environments for Children's Physical Activity (PLAYCE – Western Australia)

The PLAY Spaces & Environments for Children's Physical Activity (PLAYCE) is an ongoing cohort study (2015-current), investigating the influence of the childcare-, home-, and neighbourhood- built environments (parent perceptions and objectively measured) on children's physical activity, sedentary behaviour, diet and weight status.[32] Baseline data were collected for 2,028 children, aged 2-5 years, recruited from 118 childcare centres (response rate 46%) in metropolitan Perth, Western Australia. Childcare centres were recruited evenly across socio-economic statuses and size (small and large). Two additional data time points were collected once children commenced formal schooling, at 5-7

For peer review only - http://bmjopen.bmj.com/site/about/guidelines.xhtml

years (2019-20) and 7-9 years (2020-22). The study design enables modifiable risk factors to be examined as children transition between different built environment settings: home, childcare; and full-time school. Full details of the PLAYCE study protocol have been published.[33]

Children's height, weight and waist circumference were measured by the research team using standard protocols. Physical activity was measured using accelerometery, and parent-report structured and unstructured physical activity including outdoor play.[33–36] Sedentary behaviour was measured using accelerometery (time spent sedentary) and parent-report screen time.[33–35] Diet was assessed using a parent-report short food frequency questionnaire.[37]

The ORIGINS Project (Western Australia)

ORIGINS is a collaborative initiative between the Telethon Kids Institute and Joondalup Health Campus. It is a birth cohort of 10,000 families enrolled during pregnancy and followed children from birth to age five.[38] ORIGINS is fully integrated into clinical and diagnostic services. It aims to improve understanding of the early antecedent pathways that mediate NCDs development through the study of early environments, maternal and paternal physical health, and genetics. ORIGINS overarching goal is to reduce the rising epidemic of NCDs by providing children with 'a healthy start to life.' Data collected from active participants aged one-year old (2018-2020) and three-years old (2020-2022) be included (n=2,036).Children's height, weight and waist circumference were measured by trained staff. Physical activity was measured using parent-report structured and unstructured physical activity as well as outdoor play.[36] Sedentary behaviour was measured via parent-report screen time. Parents reported their child's diet using the Australian Eating Survey; a validated semi-quantitative food frequency questionnaire.[39]

Longitudinal Study of Australian Children (LSAC - Australia)

Growing Up in Australia: The Longitudinal Study of Australian Children (LSAC) is an ongoing prospective study that used a two cohort cross-sequential design and in 2004 recruited 5,107 children to the baby cohort (~ 9 months) and 4,983 children to the kindergarten cohort (~4.8 years).[40] The sampling method provided a representative sample of Australian children. Data were collected every two years for both cohorts. Data from children aged 8-17 years (8-9; 10-11; 12-13; 14-15; and 16-17 years old) from the Perth, Sydney and Melbourne metropolitan areas, collected between 2008 and 2018, will be included (n=3,645). Full details of the LSAC study are published elsewhere.[40]

Children's height, weight, body composition, and waist circumference were measured by parents. Physical activity was measured using parent-report time-use diaries which included structured and unstructured physical activity. Sedentary behaviour was gathered through parent reports and the children's time use diary for three types of screen-based activities (television viewing, computer use

For peer review only - http://bmjopen.bmj.com/site/about/guidelines.xhtml

and electronic games).[41] Each parents reported their child's diet via food diaries. In the Child Health CheckPoint, children completed a questionnaire regarding their usual intake of various foods including fruit and vegetable consumption, water intake, and breakfast consumption.

Data Linkage

Wales

Routinely collected health data and cohort study data from studies such as the Millennium Cohort Study, are available via the Secure Anonymised Information Linkage (SAIL) databank.[42,43] This is a unique resource of linked longitudinal health, socio-economic and environmental data relating to the people of Wales. One of the core linkage mechanisms is the Residential Anonymised Linking Field (RALF).[44] The benefits of the RALF system are three-fold. First, it allows the GIS modelling of population-level socio-environmental measures for approximately 1.4 million households in Wales at ±1m spatial resolution. Second, it allows the grouping of individuals into household units in SAIL; thus, reducing ecological fallacies and the impacts of the modifiable areal unit problem. Thirdly, it allows house moves to be identified and hence length of exposure to the built environment to be examined.

Australia

Home addresses will be geocoded for all three Australian cohorts (ORIGINS, PLAYCE and LSAC) to enable the creation of GIS measures of the natural and built environment by the Australian team.[33,45] PLAYCE and ORIGINS cohort data will be linked to built environment measures using a unique participant identification number. Where possible, change in addresses over time will be examined for these cohorts. LSAC data linkage will involve the Australian Bureau of Statistics, the Australian Institute of Family Studies' Data Linkage and Integrating Authority, the Australian Institute of Family Studies' Data Management team, National Centre for Longitudinal Data.

Statistical analysis

A series of covariate-adjusted multi-level regression models will be fitted to explore the impact of different aspects of the built environment on BMI/NCD risk factors, to quantify the effect of each associative relationship using a common outcome variable. A multi-level model structure will be used to adjust for inherent autocorrelation that exists within groups of individuals. Covariates will include, for example, child age, sex, socio-economic status (e.g., parent education, neighbourhood level disadvantage), and family composition. We will also examine how these relationships vary by different population groups (e.g., young children, school age children and adolescents). This series of analyses

For peer review only - http://bmjopen.bmj.com/site/about/guidelines.xhtml

will enable a comparison and prioritisation of the built environment factors most strongly associated with BMI and quantify the magnitude of variation in effect size across various built environment features. We will then standardise these results to rank characteristics of the built environment which may contribute to better health, to assist prioritisation of public services and resources.

The direct, indirect, and total effects of the built environment on children's BMI and other NCD risk factors will be estimated using well established multi-level mediation modelling. We will also determine if differences in these factors between advantaged and disadvantaged neighbourhoods can be explained in part by differences in built environment attributes (e.g., better or poorer access to green space). The mediation effect of physical activity and the moderation effect of neighbourhood socio-economic disadvantage on the relationship between the built environment and BMI will initially be tested as separate models. A moderated path analysis using all longitudinal data will combine moderation and mediation, and identify direct, indirect, and total mediation effects, and show how these effects vary across the levels of the moderator. All analyses will adjust for baseline and, where appropriate, temporal confounding. Special attention will also be paid to potential confounders of the outcome (e.g., BMI) and the mediating variable (e.g., physical activity) being examined. The role of age, sex, socio-economic status and urban/rural locality will be examined.

Statistical analyses will be conducted separately for Welsh and Australian datasets. A set of built environment measures will be standardised and linked to the five cohort datasets. Data governance prohibits pooling of datasets; however, findings from both countries' analysis of their respective linked health cohort and built environment datasets will be compared to determine differences between different age groups of children in Wales and Australia.

Formal statistical analysis plans will be drafted in advance of the analyses and agreed with our Expert Advisory Group. These will specify our modelling strategies, rules on dealing with missing data, and reporting conventions, including the provision of CONSORT diagrams.

Patient and Public Involvement

Development of the research questions and outcome measures were informed by ongoing engagement with stakeholders who have an interest in child health, such as Public Health Wales, and the PLAYCE Community Reference Group (Western Australia). This study uses anonymised routinely collected and cohort data, so it was not possible to directly consult the individuals included in the study.

For peer review only - http://bmjopen.bmj.com/site/about/guidelines.xhtml

Results will be disseminated by engaging children directly such as via a short video that can be shown in schools and online, science outreach workshops, and existing child-health networks to translate the findings into useful advice for children, their families, and policy makers. Please refer to 'Ethics & Dissemination' below further information.

Ethics & Dissemination

All data will be anonymised and, in Wales, linked within the privacy protecting SAIL Databank. We will be using anonymised data and therefore we are exempt from National Research Ethics Committee (NREC). An Information Governance Review Panel (IGRP) [Project ID: 1001] provided approval to link these data. The PLAYCE study has existing Human Research Ethics Approval from The University of Western Australia (UWA: 2020/ET000353). Ethics approvals have been received from Ramsay Human Research Ethics Committee Western Australia for the ORIGINS and built environment analyses (HREC 2155W) and the UWA Human Research Ethics Committee for the LSAC and built environment secondary analyses (2022/ET000573). All Australian research will abide by the NHMRC's National Statement on Ethical Conduct in Human Research (2008) and is considered negligible risk.

Our dissemination and impact generation strategies will be informed by research on knowledge mobilization models; [46] to impact at a variety of scales (including national and local government and across multiple sectors) and will not be confined to a Welsh-Australian context. Throughout, we will involve stakeholders and policy makers in the research process from inception to translation, seeking their feedback on our process, progress, and findings. We will disseminate findings through community engagement (e.g., at public events for families or to school management teams), and through existing and new industry partner networks. An information sheet will be prepared in plain English with infographics, and a video explaining the project and its findings will be made available online and advertised through social media. Findings from the study will be published in a comprehensive report and an infographic summary. We will notify all stakeholders and promote publication through international networks established during this research. We will hold seminars and workshops to report our findings to stakeholders and the public.

For peer review only

Discussion

The purpose of this study is to identify and understand how complex and interacting factors in the built environment influence modifiable risk factors for NCDs across childhood. Harmonising both child and built environment indicators across five large-scale studies enables analysis of an effectively larger sample size, spans a broader set of age-ranges, and leverages greater heterogeneity in built environments. By harmonising analyses from Welsh and Australian settings, the BEACHES project provides a unique opportunity to identify impacts of the built environment that are common across settings and where place-specific, physical, cultural, and policy environments mediate these effects.

We aim to communicate to policy and planners which modifiable aspects of the built environment result in the most significant reductions in the risk factors for NCDs across childhood. This will enable action at both a national and international scale and, in turn, will contribute to population-level reductions in NCDs. This is an essential component of our work to able to bring about large-scale improvement in the built environment to reduce NCDs beginning in early childhood. Furthermore, the outputs of this research could be used to inform future research by aggregating evidence of individual associations in a single dynamic model.

Contributorship statement

HC, GS, LG, and RF are the Principal Investigators who conceptualised the overall study, established research collaborations and led the project implementation. Co-Investigators who contributed to obtaining the research funding include BB (Beck), BB (Boruff), SB, DC, PG, RAL, AM, KM, MR, JS, ST, AW. These Principle and Co-Investigators, in additional other team members (RP-C, RB, BB (Beesley), JD, RDJ, JR, DAT, TR) are responsible for the development of Geographic Information Systems measures and associated data analysis. RB, KM, AW, RDJ, JR, HC and LJG are responsible for the design and implementation of the statistical analyses. GS, TP, HC, GD are responsible for the policy reviews and stakeholder engagement. RP-C, GD, RF, HC, and LJG, drafted this manuscript. All authors revised and approved the final version of the manuscript.

For peer review only - http://bmjopen.bmj.com/site/about/guidelines.xhtml

Competing interests

No competing interests to declare.

Funding

This Welsh arm of the BEACHES study was funded by the UK Medical Research Council (MRC) grant number MR/T039329/1. This work will also be supported by the Administrative Data Research (ADR) Wales, part of the ADR UK investment, which unites research expertise from Swansea University Medical School and WISERD (Wales Institute of Social and Economic Research and Data) at Cardiff University with analysts from Welsh Government. ADR UK is funded by the Economic and Social Research Council (ESRC), part of UK Research and Innovation. The Millennium Cohort Study is funded by grants to the Centre for Longitudinal Studies at the Institute of Education from the Economic and Social Research Council and a consortium of government department.

The Australian arm of the BEACHES study was funded by the Australian National Health and Medical Research Council - UKRI-NHMRC Built Environment Prevention Research Scheme (GNT1192764). This research is supported (partially) by the Australian Government through the Australian Research Council's Centre of Excellence for Children and Families over the Life Course (Project ID CE200100025). HC is supported by an Australian National Heart Foundation Future Leader Fellowship (102549). The ORIGINS Project has received core funding support from the Telethon Perth Children's Hospital Research Fund, Joondalup Health Campus, the Paul Ramsay Foundation and the Commonwealth Government of Australia through the Channel 7 Telethon Trust. Substantial in-kind support has been provided by Telethon Kids Institute and Joondalup Health Campus.

References

1 Bentham J, Di Cesare M, Bilano V, et al. Worldwide trends in body-mass index, underweight, overweight, and obesity from 1975 to 2016: a pooled analysis of 2416 population-based measurement studies in 128·9 million children, adolescents, and adults. *Lancet*. 2017;390(10113).

2 WHO. Report of the Commission on Ending Childhood Obesity: implementation plan Report by the Secretariat [Internet]. 2017. Available from: http://apps.who.int/iris/bitstream/10665/204176/1/9789241510066_eng.pdf?ua=1

For peer review only - http://bmjopen.bmj.com/site/about/guidelines.xhtml

3 Coppinger T, Milton K, Murtagh E, et al. Global Matrix 3.0 physical activity report card for children and youth: a comparison across Europe. *Public Health*. 2020;187:150–6.

4 Christian HE, Bull FC, Middleton NJ, et al. How important is the land use mix measure in understanding walking behaviour? Results from the RESIDE study. *Int J Behav Nutr Phys Act*. 2011;8(1):55.

5 Foster S, Wood L, Christian H, et al. Planning safer suburbs: Do changes in the built environment influence residents' perceptions of crime risk? *Soc Sci Med*. 2013;97:87–94.

6 Christian H, Knuiman M, Divitini M, et al. A Longitudinal Analysis of the Influence of the Neighborhood Environment on Recreational Walking within the Neighborhood: Results from RESIDE. *Environ Health Perspect*. 2017;125(7):077009.

7 Müller-Riemenschneider F, Pereira G, Villanueva K, et al. Neighborhood walkability and cardiometabolic risk factors in australian adults: An observational study. *BMC Public Health*. 2013;13(1):755.

8 Bird EL, Ige JO, Pilkington P, et al. Built and natural environment planning principles for promoting health: An umbrella review. *BMC Public Health*. 2018;18(1).

9 Ortegon-Sanchez A, McEachan RRC, Albert A, et al. Measuring the Built Environment in Studies of Child Health-A Meta-Narrative Review of Associations. *Int J Environ Res Public Health*. 2021;18(20).

10 Nobles J, Summerbell C, Brown T, et al. A secondary analysis of the childhood obesity prevention Cochrane Review through a wider determinants of health lens: implications for research funders, researchers, policymakers and practitioners. *Int J Behav Nutr Phys Act*. 2021;18(1).

11 Dixon BN, Ugwoaba UA, Brockmann AN, et al. Associations between the built environment and dietary intake, physical activity, and obesity: A scoping review of reviews. *Obes Rev*. 2021;22(4):e13171.

12 Jia P, Zou Y, Wu Z, et al. Street connectivity, physical activity, and childhood obesity: A systematic review and meta-analysis. *Obes Rev*. 2021;22(S1):e12943.

13 Volger S, Rigassio Radler D, Rothpletz-Puglia P. Early childhood obesity prevention efforts through a life course health development perspective: A scoping review. Ells L, editor. *PLoS One*. 2018;13(12):e0209787.

For peer review only

For peer review only - http://bmjopen.bmj.com/site/about/guidelines.xhtml

14 Wu S, Ding Y, Wu F, et al. Socio-economic position as an intervention against overweight and obesity in children: A systematic review and meta-analysis. *Sci Rep*. 2015;5.

15 Lobstein T, Jackson-Leach R, Moodie ML, et al. Child and adolescent obesity: Part of a bigger picture [Internet]. Vol. 385, The Lancet. Lancet Publishing Group; 2015. p. 2510–20.

16 Blüher M. Obesity: global epidemiology and pathogenesis [Internet]. Vol. 15, Nature Reviews Endocrinology. Nature Publishing Group; 2019. p. 288–98.

17 Coghlan AT, Preskill H, Tzavaras Catsambas T. An overview of appreciative inquiry in evaluation. *New Dir Eval*. 2003;2003(100):5–22.

18 Cooperrider DL, Whitney DK. Appreciative inquiry : a positive revolution in change [Internet]. . ReadHowYouWant. com; 111 p. Available from: https://books.google.com/books/about/Appreciative_Inquiry.html?id=bJBgktz2Oa8C

19 Public Health Wales. Creating healthier places and spaces for our present and future generations [Internet]. 2018. Available from: www.nationalarchives.gov.uk/doc/open-government-licence/version/3/

20 Klepac Pogrmilovic B, O'Sullivan G, Milton K, et al. The development of the Comprehensive Analysis of Policy on Physical Activity (CAPPA) framework. *Int J Behav Nutr Phys Act*. 2019;16(1):1–15.

21 Mizen A, Song J, Fry R, et al. Longitudinal access and exposure to green-blue spaces and individual-level mental health and well-being: Protocol for a longitudinal, population-wide record-linked natural experiment. *BMJ Open*. 2019;9(4).

22 Caccetta P, Collings S, Devereux A, et al. GOVERNMENT SERVICES, CSIRO DIGITAL PRODUCTIVITY FLAGSHIP Urban Monitor: Enabling effective monitoring and management of urban and coastal environments using digital aerial photography Final Report-Transformation of aerial photography into digital raster information products. 2012;

23 Mavoa S, Bagheri N, Koohsari MJ, et al. How Do Neighbourhood Definitions Influence the Associations between Built Environment and Physical Activity? *Int J Environ Res Public Health*. 2019;16(9):1501.

24 Noardo F, Lingua A, Aicardi I, et al. Cartographic data harmonisation for a cross-border project development. *Appl Geomatics*. 2016;8(3):133–50.

25 Fry R, Orford S, Rodgers S, et al. A best practice framework to measure spatial variation in

For peer review only

For peer review only - http://bmjopen.bmj.com/site/about/guidelines.xhtml

alcohol availability. *Environ Plan B Urban Anal City Sci*. 2020;47(3):381–99.

26 Hyatt M, Rodgers SE, Paranjothy S, et al. The wales electronic cohort for children (WECC) study. *Arch Dis Child - Fetal Neonatal Ed*. 2011;96(Suppl 1):Fa18–Fa18.

27 Connelly R, Platt L. Cohort profile: UK Millennium Cohort Study (MCS). *Int J Epidemiol*. 2014;43(6):1719–25.

28 Fitzsimons E BD. Obesity prevalence and its inequality from childhood to adolescence [Internet]. Available from: https://cls.ucl.ac.uk/wp-content/uploads/2020/12/CLS-briefing-paper-Obesity-prevalence-MCS-age-17-initial-findings.pdf

29 Griffiths LJ, Cortina-Borja M, Sera F, et al. How active are our children? Findings from the Millennium Cohort Study. *BMJ Open*. 2013;3(8):e002893.

30 Fluharty M, Villadsen A, Kandola A, et al. Physical activity across age and study: a guide to data in six CLOSER studies Resource report. 2020.

31 Maddock J, O'neill D, Robinson S, et al. A guide to the dietary data in eight CLOSER studies Resource report. 2020.

32 Christian H, Rosenberg M, Trost S, et al. A snapshot of the PLAYCE project: Findings from the Western Australian PLAY Spaces and Environments for Children's Physical Activity Study. Supportive Childcare Environments for Physical Activity in the Early Years. Perth, Western Australia: The Universit. 2018 Dec.

33 Christian H, Maitland C, Enkel S, et al. Influence of the day care, home and neighbourhood environment on young children's physical activity and health: protocol for the PLAYCE observational study. *BMJ Open*. 2016;6(12):14058.

34 Pate RR, O'Neill JR, Mitchell J. Measurement of physical activity in preschool children. *Med Sci Sports Exerc*. 2010;42(3):508–12.

35 Hinkley T, Salmon J, Okely AD, et al. The HAPPY study: development and reliability of a parent survey to assess correlates of preschool children's physical activity. *J Sci Med Sport*. 2012;15(5):407–17.

36 Burdette HL, Whitaker RC, Daniels SR. Parental report of outdoor playtime as a measure of physical activity in preschool-aged children. *Arch Pediatr Adolesc Med*. 2004;158(4):353–7.

37 Hendrie GA, Viner Smith E, Golley RK. The reliability and relative validity of a diet index score for 4-11-year-old children derived from a parent-reported short food survey. *Public Health*

For peer review only - http://bmjopen.bmj.com/site/about/guidelines.xhtml

Nutr. 2014;17(7):1486–97.

38 Silva DT, Hagemann E, Davis JA, et al. Introducing the ORIGINS project: a community-based interventional birth cohort. *Rev Environ Health*. 2020;35(3):281–93.

39 Collins CE, Burrows TL, Truby H, et al. Comparison of energy intake in toddlers assessed by food frequency questionnaire and total energy expenditure measured by the doubly labeled water method. *J Acad Nutr Diet*. 2013;113(3):459–63.

40 Soloff C, Lawrence D, Misson S, et al. The Longitudinal Study of Australian Children: an Australian Government initiative - Wave 1 weighting and non-response. 2006.

41 Yu M, Baxter J. 5. Australian children’s screen time and participation in extracurricular activities - LSAC Annual Statistical Report 2015.

42 Lyons RA, Jones KH, John G, et al. The SAIL databank: Linking multiple health and social care datasets. *BMC Med Inform Decis Mak*. 2009;9(1):3.

43 Ford D V., Jones KH, Verplancke JP, et al. The SAIL Databank: Building a national architecture for e-health research and evaluation. *BMC Health Serv Res*. 2009;9(1):157.

44 Johnson RD, Griffiths LJ, Hollinghurst JP, et al. Deriving household composition using population-scale electronic health record data—A reproducible methodology. *PLoS One*. 2021;16(3):e0248195.

45 Hooper P, Foster S, Bull F, et al. Living liveable? RESIDE’s evaluation of the “Liveable Neighborhoods” planning policy on the health supportive behaviors and wellbeing of residents in Perth, Western Australia. *SSM - Popul Heal*. 2020;10:100538.

46 Davies HT, Powell AE, Nutley SM. Mobilising knowledge to improve UK health care: learning from other countries and other sectors – a multimethod mapping study. *Heal Serv Deliv Res*. 2015;3(27):1–190.

Figure Legend

Figure 1 - BEACHES project workflow.

For peer review only

Additional Information

Acknowledgements

This study makes use of anonymised data held in the Secure Anonymised Information Linkage (SAIL) Databank. We would like to acknowledge all the data providers who make anonymised data available for research. We also thank all the Millennium Cohort families for their participation, the director of the Millennium Cohort Study and colleagues in the management team at the Centre for Longitudinal Studies, UCL Institute of Education. Partners for the Australian arm include the Western Australian (WA) Department of Local Government, Sport and Cultural Industries, WA Department of Health, WA Department of Transport, WA Local Government Association, Australian Childcare Alliance, Nature Play Australia, Heart Foundation, Cancer Council WA, Goodstart Early Learning, and Hames Sharley. The ORIGINS Project is only possible because of the commitment of the families in ORIGINS. We are grateful to all the participants, health professionals and researchers who support the project. We would also like to acknowledge and thank the following teams and individuals who have made The ORIGINS Project possible: The ORIGINS Project team; CEO Dr Kempton Cowan, executive staff and obstetric, neonatal and paediatric teams, Joondalup Health Campus (JHC); Director Professor Jonathan Carapetis and executive staff, Telethon Kids Institute; Mayor Tracey Roberts, City of Wanneroo; Mayor Albert Jacobs, City of Joondalup; Professor Fiona Stanley, patron of ORIGINS; members of ORIGINS Community Reference and Participant Reference Groups; Research Interest Groups and the ORIGINS Scientific Committee. BEACHES is a sub-project of The ORIGINS Project. This unique long-term study, a collaboration between Telethon Kids Institute and Joondalup Health Campus, is one of the most comprehensive studies of pregnant women and their families in Australia to date, recruiting 10,000 families over a decade from the Joondalup and Wanneroo communities of Western Australia.

Disclaimer

The views expressed in this paper are those of the authors and not necessarily those of the funding bodies, the NHS, The NIHR, or the Department of Health and Social Care.

Acronyms

For peer review only

BMI	Body Mass Index
CMPW	Child Measurement Programme for Wales
EVI	Enhanced Vegetation Index
GIS	Geographic Information Systems
GNAF	Geoscape Geocoded National Address File
GP	General Practitioner
LSAC	Longitudinal Study of Australian Children
MCS	Millennium Cohort Study
NCD	Non-Communicable Disease
NDVI	Normalised Difference Vegetation Index
ONS	Office for National Statistics
OS	Ordnance Survey
OSM	Open Street Map
PLAYCE	PLAY Spaces & Environments for Children's Physical Activity
RALF	Residential Anonymised Linking Field
SAIL	Secure Anonymised Information Linkage
SES	Socio-economic status
WA	Western Australia
WECC	Wales Electronic Cohort for Children
UK	United Kingdom
UPRN	Unique Property Reference Number

For peer review only - http://bmjopen.bmj.com/site/about/guidelines.xhtml

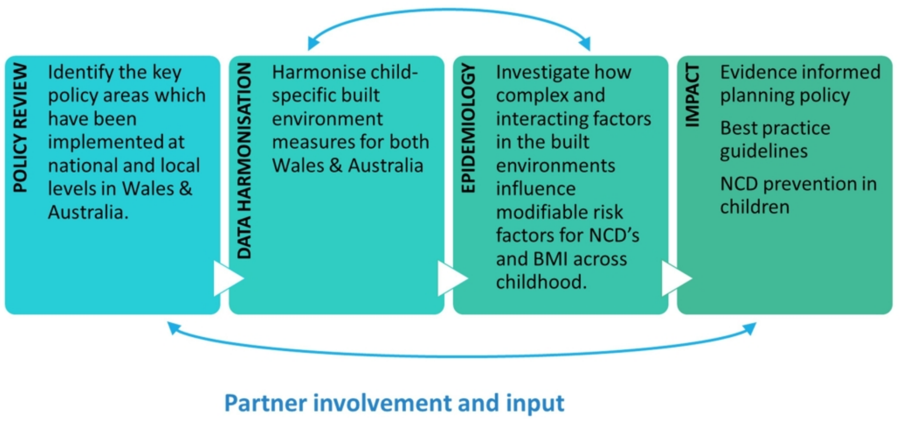

BEACHES project workflow

297x209mm (300 x 300 DPI)

bmjopen-2022-061978 - "Built Environment and Child Health in Wales and Australia (BEACHES): a study protocol"

STROBE Statement—checklist of items that should be included in reports of observational studies

	Item No.	Recommendation	Page No.	Relevant text from manuscript
**Title and abstract**	1	(*a*) Indicate the study's design with a commonly used term in the title or the abstract	1<br>3	Title - Line: 1<br>Abstract - Lines: 67
		(*b*) Provide in the abstract an informative and balanced summary of what was done and what was found	3	Lines: 67-74<br>As this is a protocol, we also report how findings will be disseminated in lines 79-83
**Introduction**				
Background/rationale	2	Explain the scientific background and rationale for the investigation being reported	4-5	Lines 101-136
Objectives	3	State specific objectives, including any prespecified hypotheses	5-6	Lines: 139-152
**Methods**				
Study design	4	Present key elements of study design early in the paper	6	Lines: 156-166
Setting	5	Describe the setting, locations, and relevant dates, including periods of recruitment, exposure, follow-up, and data collection	6/10	Lines: 156-166; Table 2
Participants	6	(*a*) *Cohort study*—Give the eligibility criteria, and the sources and methods of selection of participants. Describe methods of follow-up<br>*Case-control study*—Give the eligibility criteria, and the sources and methods of case ascertainment and control selection. Give the rationale for the choice of cases and controls<br>*Cross-sectional study*—Give the eligibility criteria, and the sources and methods of selection of participants	10-12	Description of cohort data<br>Lines 245-326
		(*b*) *Cohort study*—For matched studies, give matching criteria and number of exposed and unexposed<br>*Case-control study*—For matched studies, give matching criteria and the number of controls per case	n/a	
Variables	7	Clearly define all outcomes, exposures, predictors, potential confounders, and effect modifiers. Give diagnostic criteria, if applicable	13	Covariates - Lines: 352-354

For peer review only - http://bmjopen.bmj.com/site/about/guidelines.xhtml

For peer review only

Data sources/ measurement	8*	For each variable of interest, give sources of data and details of methods of assessment (measurement). Describe comparability of assessment methods if there is more than one group	7-9<br><br>10-12	Built Environment - Lines:200-239<br>Health data - Lines: 245-326
Bias	9	Describe any efforts to address potential sources of bias	n/a	
Study size	10	Explain how the study size was arrived at	10	Table 2 details cohort sizes
Quantitative variables	11	Explain how quantitative variables were handled in the analyses. If applicable, describe which groupings were chosen and why	n/a	
Statistical methods	12	(*a*) Describe all statistical methods, including those used to control for confounding	13<br>13-14	Data linkage - Lines:328-346<br>Statistical analyses - Lines: 348-382
		(*b*) Describe any methods used to examine subgroups and interactions		
		(*c*) Explain how missing data were addressed	n/a	
		(*d*) *Cohort study*—If applicable, explain how loss to follow-up was addressed<br>*Case-control study*—If applicable, explain how matching of cases and controls was addressed<br>*Cross-sectional study*—If applicable, describe analytical methods taking account of sampling strategy	n/a	
		(*e*) Describe any sensitivity analyses	13	Analysis of different population groups - Lines: 354-355
**Results**				
Participants	13*	(a) Report numbers of individuals at each stage of study—eg numbers potentially eligible, examined for eligibility, confirmed eligible, included in the study, completing follow-up, and analysed	n/a	
		(b) Give reasons for non-participation at each stage	n/a	
		(c) Consider use of a flow diagram	n/a	
Descriptive data	14*	(a) Give characteristics of study participants (eg demographic, clinical, social) and information on exposures and potential confounders	n/a	
		(b) Indicate number of participants with missing data for each variable of interest	n/a	
		(c) *Cohort study*—Summarise follow-up time (eg, average and total amount)	n/a	
Outcome data	15*	*Cohort study*—Report numbers of outcome events or summary measures over time	n/a	

For peer review only

		Case-control study—Report numbers in each exposure category, or summary measures of exposure	n/a	
		Cross-sectional study—Report numbers of outcome events or summary measures		
Main results	16	(*a*) Give unadjusted estimates and, if applicable, confounder-adjusted estimates and their precision (eg, 95% confidence interval). Make clear which confounders were adjusted for and why they were included	n/a	
		(*b*) Report category boundaries when continuous variables were categorized	n/a	
		(*c*) If relevant, consider translating estimates of relative risk into absolute risk for a meaningful time period	n/a	
Other analyses	17	Report other analyses done—eg analyses of subgroups and interactions, and sensitivity analyses	n/a	
**Discussion**				
Key results	18	Summarise key results with reference to study objectives	n/a	
Limitations	19	Discuss limitations of the study, taking into account sources of potential bias or imprecision. Discuss both direction and magnitude of any potential bias	4	Strengths and limitations of the study – Lines: 87-98
Interpretation	20	Give a cautious overall interpretation of results considering objectives, limitations, multiplicity of analyses, results from similar studies, and other relevant evidence	n/a	
Generalisability	21	Discuss the generalisability (external validity) of the study results	15-16	Discussion - Lines: 421-437
**Other information**				
Funding	22	Give the source of funding and the role of the funders for the present study and, if applicable, for the original study on which the present article is based	16	Funding - Lines: 453-469

*Give information separately for cases and controls in case-control studies and, if applicable, for exposed and unexposed groups in cohort and cross-sectional studies.

Note: An Explanation and Elaboration article discusses each checklist item and gives methodological background and published examples of transparent reporting. The STROBE checklist is best used in conjunction with this article (freely available on the Web sites of PLoS Medicine at http://www.plosmedicine.org/, Annals of Internal Medicine at http://www.annals.org/, and Epidemiology at http://www.epidem.com/). Information on the STROBE Initiative is available at www.strobe-statement.org.

For peer review only
